# Visualising lead optimisation series using reduced graphs

**DOI:** 10.1186/s13321-025-01002-7

**Published:** 2025-04-24

**Authors:** Jessica Stacey, Baptiste Canault, Stephen D. Pickett, Valerie J. Gillet

**Affiliations:** 1 Information School, University of Sheffield, The Wave, 2 Whitham Road, Sheffield, S10 2AH UK; 2 GlaxoSmithKline, Gunnels Wood Road, Stevenage, Herts SG1 2NY UK

**Keywords:** Reduced graphs, Visualisation, Lead optimisation, SAR

## Abstract

The typical way in which lead optimisation (LO) series are represented in the medicinal chemistry literature is as Markush structures and associated R-group tables. The Markush structure shows a central core or molecular scaffold that is common to the series with R groups that indicate the points of variability that have been explored in the series. The associated R-group table shows the substituent combinations that exist in individual molecules in the series together with properties of those compounds. This format provides an intuitive way of visualising any structure–activity relationship (SAR) that is present. Automated approaches that attempt to reproduce this well understood format, such as the SAR map, are based on maximum common substructure approaches and do not take account of small changes that may be made to the core structure itself or of the situation where more than one core exists in the data. Here we describe an automated approach to represent LO series that is based on reduced graph descriptions of molecules. A publicly available LO dataset from a drug discovery programme at GSK is analysed to show how the method can group together compounds from the same series even when there are small substructural differences within the core of the series while also being able to identify different related compound series. The resulting visualisation is useful in identifying areas where series are under explored and for mapping design ideas onto the current dataset. The code to generate the visualisations is released into the public domain to promote further research in this area.

**Scientific contribution**: We describe a software tool for analysing lead optimisation series using reduced graph representations of molecules. The representation allows compounds that have similar but not identical chemical scaffolds to be grouped together and is, therefore, an advance on methods that are based on the more traditional Markush structure and SAR tables. The software is a useful addition to the med chem toolbox as it can provide a holistic view of lead optimisation data by representing what might otherwise be seen as separate series as a single series of compounds.

**Supplementary Information:**

The online version contains supplementary material available at 10.1186/s13321-025-01002-7.

## Introduction

**Fig. 1:**
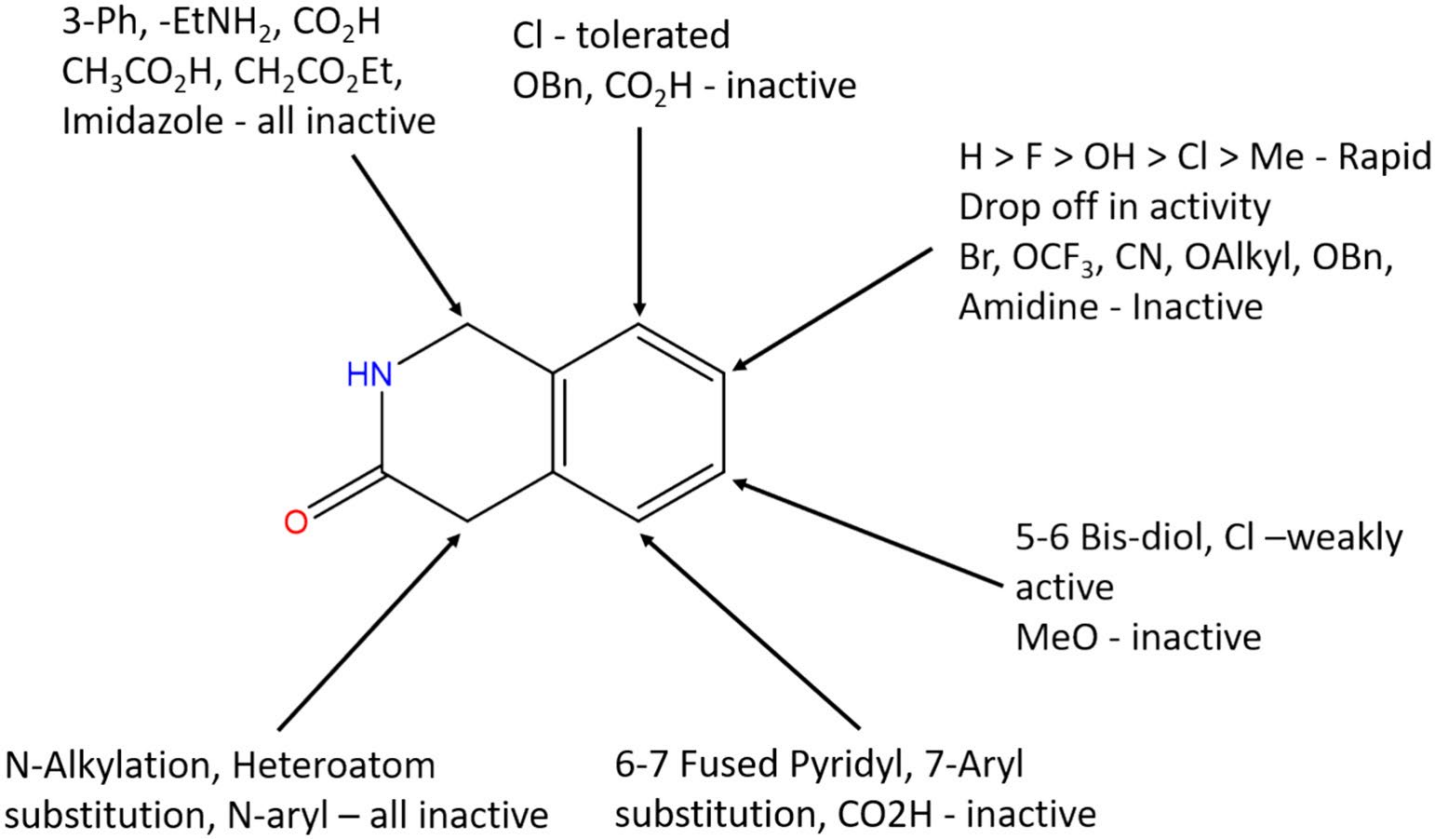
An example of a Markush representation of a lead optimisation series which shows how different substituents have been explored on a core scaffold

The aim of the lead optimisation (LO) stage of drug discovery is to improve the physicochemical property profiles of compounds that have been found to exhibit activity against a target of interest. This typically involves attempting to improve the absorption, distribution, metabolism, excretion and toxicity (ADMET) properties while retaining or improving on potency. Once a few active analogues have been identified, substituents at different positions on the molecules are modified iteratively to establish the structure–activity relationship (SAR) and identify compounds with improved properties. LO datasets, therefore, generally contain hundreds of molecules that are built around a small number of core scaffolds. An example is illustrated in Fig. 1, where the scaffold has different substitution points identified on the ring, indicated by the arrows. LO datasets are typically represented as Markush structures with associated R-group tables. A Markush structure depicts the core scaffold which is common to all members of a chemical series with the substituents shown as R groups. The associated R-group, or SAR, table is used to indicate the different substituents at each R group position for a particular molecule together with properties of the molecules [1, 2]. Markush structures and R-group tables have been widely adopted, especially in the medicinal chemistry literature, as they enable the SAR to be easily visualised.

Several different methods have been developed to automate the process of visualising SAR datasets. SAR maps are aimed at individual chemical series and aim to reproduce the Markush and SAR table described above [2]. A maximum common substructure (MCS) algorithm is used to identify the common substructure in a series of compounds which are then decomposed to identify lists of substituents and their attachment points to the common substructure. The substituent lists for two positions of variability are displayed as two-dimensional heatmaps (tables) with colour coding used to indicate the properties of the individual compounds. Wasseman and Bajorath extended this approach by introducing a graph structure for the display of chemical series to allow any number of substitution sites to be visualised [3]. These approaches assume a single analogue series is present. Stumpfe et al. developed a method to detect multiple series of analogues based on the concept of matched molecular pairs (MMP) which are pairs of compounds that differ by a single substituent [4]. The approach involves first generating a network by connecting compounds that represent MMP relationships. Disjoint clusters in the global network were found to represent analogues series, that cover all of the substitution sites in the series.

A variety of methods that are not specifically focused on compound series have been developed to organise and represent more heterogeneous datasets. These include methods based on the concept of molecular scaffolds which, distinct from MCS approaches, are defined according to rules that are independent of the dataset being processed [1]. The most common of these is the Murcko framework which was proposed by Bemis and Murcko [5].The Murcko framework involves removing the side chains from a molecule to leave the ring systems and any linking groups. The concept can be generalised by reducing all remaining atoms to carbon and all bonds to single bonds. The scaffolds can be used to cluster data according to distinct scaffolds at different levels of detail. Extensions of this basic approach have also been developed which allow compounds to be arranged hierarchically according to the constituent parts of their molecular scaffolds [6–8]. Other network-based approaches that are applicable to heterogeneous datasets include forming connections between pairs of molecules according to their structural similarity based on molecular fingerprints [9–11]. Finally, dimensionality reduction techniques including Principal Component Analysis (PCA), t-Distributed Stochastic Neighbor Embedding (t-SNE), Uniform Manifold Approximation and Projection (UMAP), and Generative Topographic Mapping (GTM) have been applied to chemical libraries to enable them to be plotted in 2- or 3-dimensions with their main application being to compare the coverage of different compound collections, see for example, [12].

A limitation of the MCS approaches to identify chemical series is that both the core scaffold and the substituents are represented as substructural fragments so that a small change to the core can lead to a new scaffold being produced and therefore a new Markush structure, which can then make it more challenging to interpret the SAR across the series. Here, we describe a method for representing LO series that overcomes some of the limitations of the typical Markush and R-group tables. Our approach is based on reduced graph (RG) representations of chemical structures which are insensitive to some small changes in substructures. An RG is a summary representation of a molecular structure whereby atoms are grouped into RG nodes according to node definitions which are usually based on the presence of cyclic and acyclic features and functional groups. This enables different substructures to be reduced to the same node type so that the RG representation is a many-to-one representation where multiple molecules can produce the same RG. RGs have been used in a range of chemoinformatics applications from representing and searching Markush structures in chemical patents, to identifying SAR and for scaffold-hopping [13–17]. They have also been used in the inSARa approach to provide a network-based view of a chemical dataset with compounds being connected in the network if they share an MCS at the RG level [18].

Here we describe a method for organising compounds using reduced graphs that is aimed at identifying and visualising LO series. Our method is based on identifying one or more MCS that is common to a set of compounds. As the underlying representation is the reduced graph, the approach allows molecules with closely related but not necessarily identical substructural scaffolds to be grouped into a single series. The method is also able to identify different compound series within a single dataset. We provide an illustration of the visualisation before describing the methodology in detail and its application to a publicly available dataset that represents different strands of a LO programme carried out at GSK.

### Overview of the visualisation

**Fig. 2:**
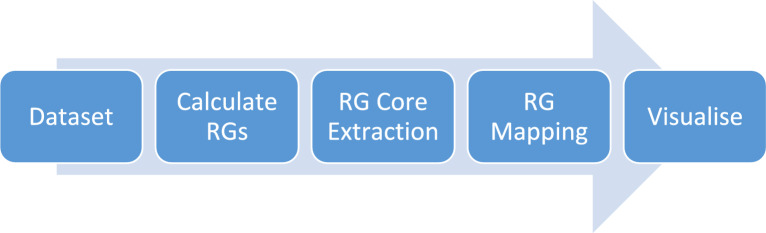
Summary of the steps involved to organise and visualise molecules

**Fig. 3:**
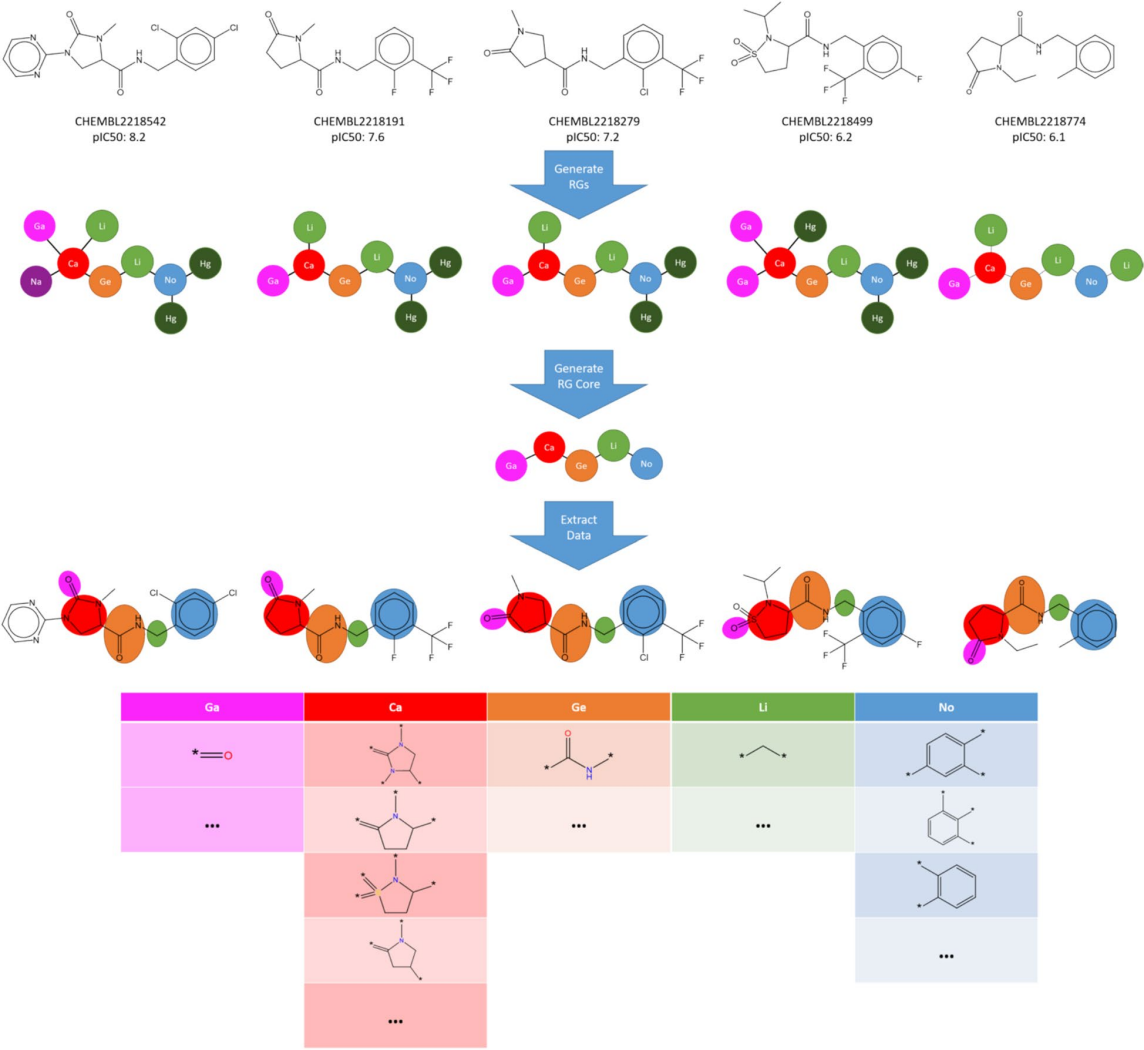
Illustration of the application of the workflow to compounds in the P2X7 dataset, which is described later

Figure 2 presents an overview of the process for generating the visualisation for a LO dataset. The details are provided below in the Methods section. In summary, the molecules in a dataset are organised according to the RG equivalent of one or more Markush structures. First, the individual molecules are represented by RGs. Next an MCS algorithm is used to identify one or more RG subgraph which is common to a set of molecules; we refer to these as RG cores. An RG core therefore provides a summary representation of a set of closely related molecules. The nodes of the RG core are annotated with the substructures they represent in the individual molecules. Thus, it is possible to drill down and visualise the different substructures that give rise to a node in the RG core. An illustrative example based on five molecules from the P2X7 dataset (described later in the Results section) is shown in Fig. 3. The molecules are first represented by RGs with the different colours and labels used to differentiate the nodes (the node types and their labels are described below). Next, the largest RG subgraph that is common to all molecules is identified and becomes the RG core. Finally, the nodes are annotated with the underlying substructures which can be visualised in tabular form as shown. For this set of molecules, the underlying substructures for three of the nodes in the RG core do not vary across the series of molecules, hence there is just a single entry in the relevant column in the table. One of the nodes represents four different substructures and another represents three different substructures. Note that substructures with different substitution patterns are identified as distinct substructures so that the node labelled “No” has three different substructure entries in the table.

**Fig. 4:**
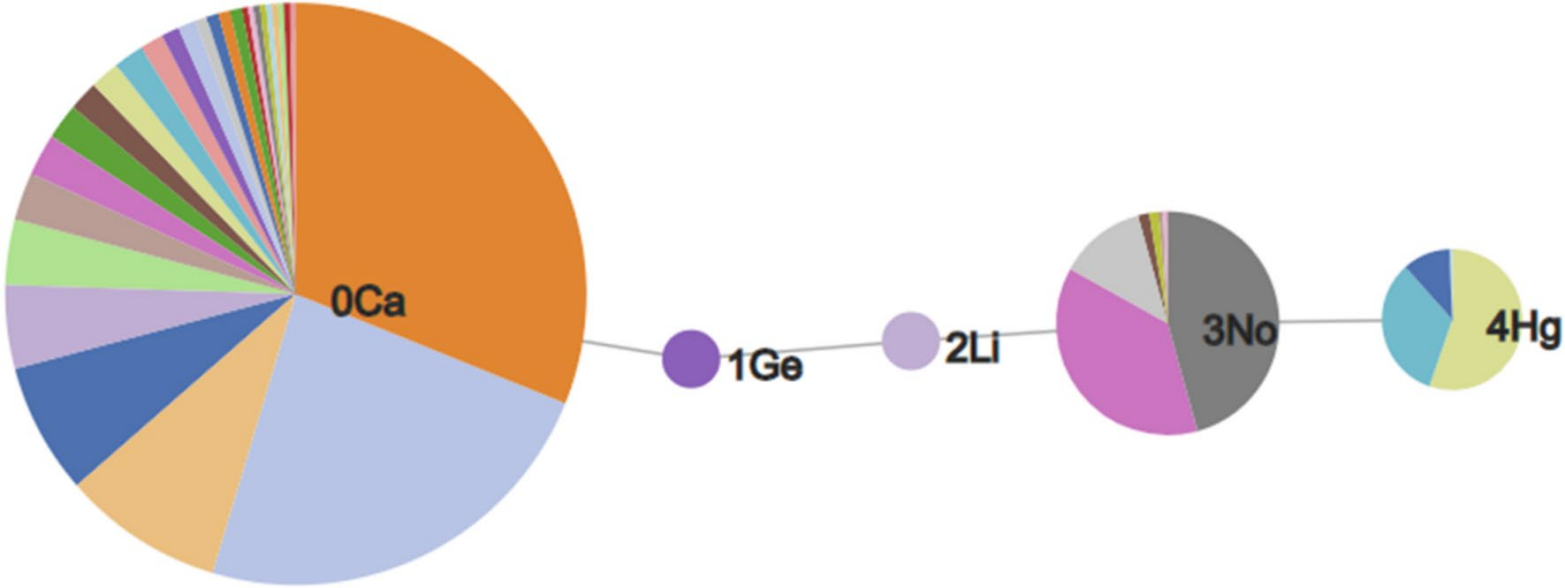
An RG core that represents 302 compounds in P2X7. The nodes are represented by text labels that specify the node type and numerical indices which allow them to be identified uniquely. The text labels correspond to elements outside of those commonly seen in organic molecules, for example, “Ca” represents an aliphatic node with hydrogen bond acceptor character. The different node types and their labels are described later. The substructures represented by the node labelled “3No” are shown in Fig. 5

**Fig. 5:**
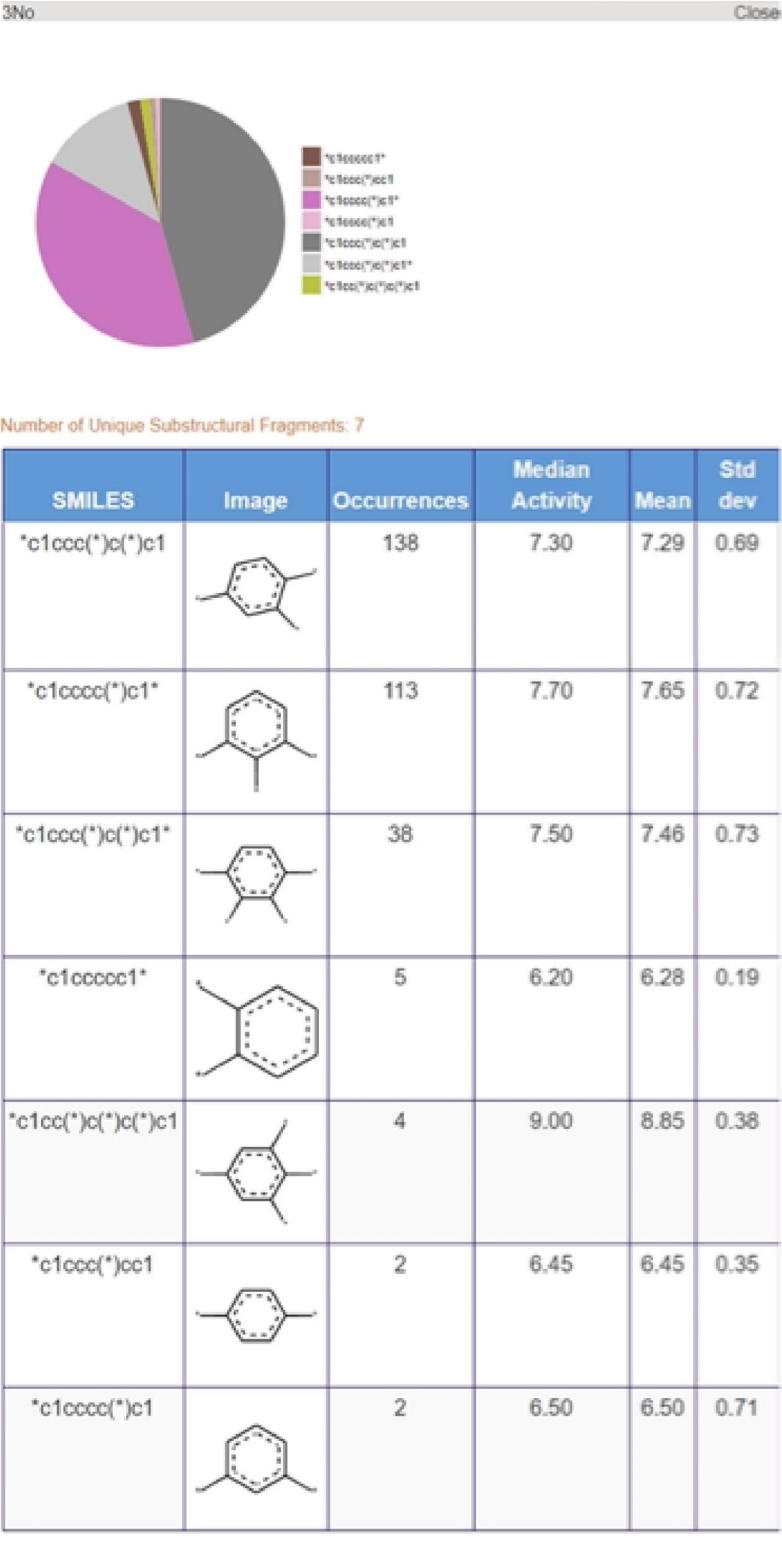
The substructures represented by No node of the RG core shown in Fig. 4

An RG core can also be represented using pie charts to display the nodes as shown in Fig. 4 where the RG core represents a set of 302 molecules identified in the P2X7 dataset. The size of each node is proportional to the number of unique substructures in the series of molecules it represents, and each node is divided into segments of size proportional to the frequency of occurrence of a given substructure in the set. In the example shown, two of the nodes (Ge and Li) each represent a single substructure which is common to all molecules in the set. The numerical labels attached to each node in the figure are simply node indices to enable the nodes to be identified uniquely. The other three nodes, Ca, No and Hg represent multiple substructures. Node Ca is the largest and represents 28 different substructures; the No node represents seven different substructures; and the Hg node represents three substructures. The depiction of the nodes of an RG core as pie charts of varying size and numbers of segments enables the extent to which substituents at those positions have been explored to be readily seen and can give an indication of regions of chemical space that have been under- and over-explored. The visualisation is interactive such that a node can be selected and a pop-up window is displayed showing a table of substructures that map to the node and the number of times that substructure occurs at that position in the set of molecules that map to the RG core, as shown in Fig. 5 The median, mean and standard deviation of the pIC50 activity data is also reported for those molecules that contain that substructure, in order to indicate the effect of each substructure on the activity.

**Fig. 6:**
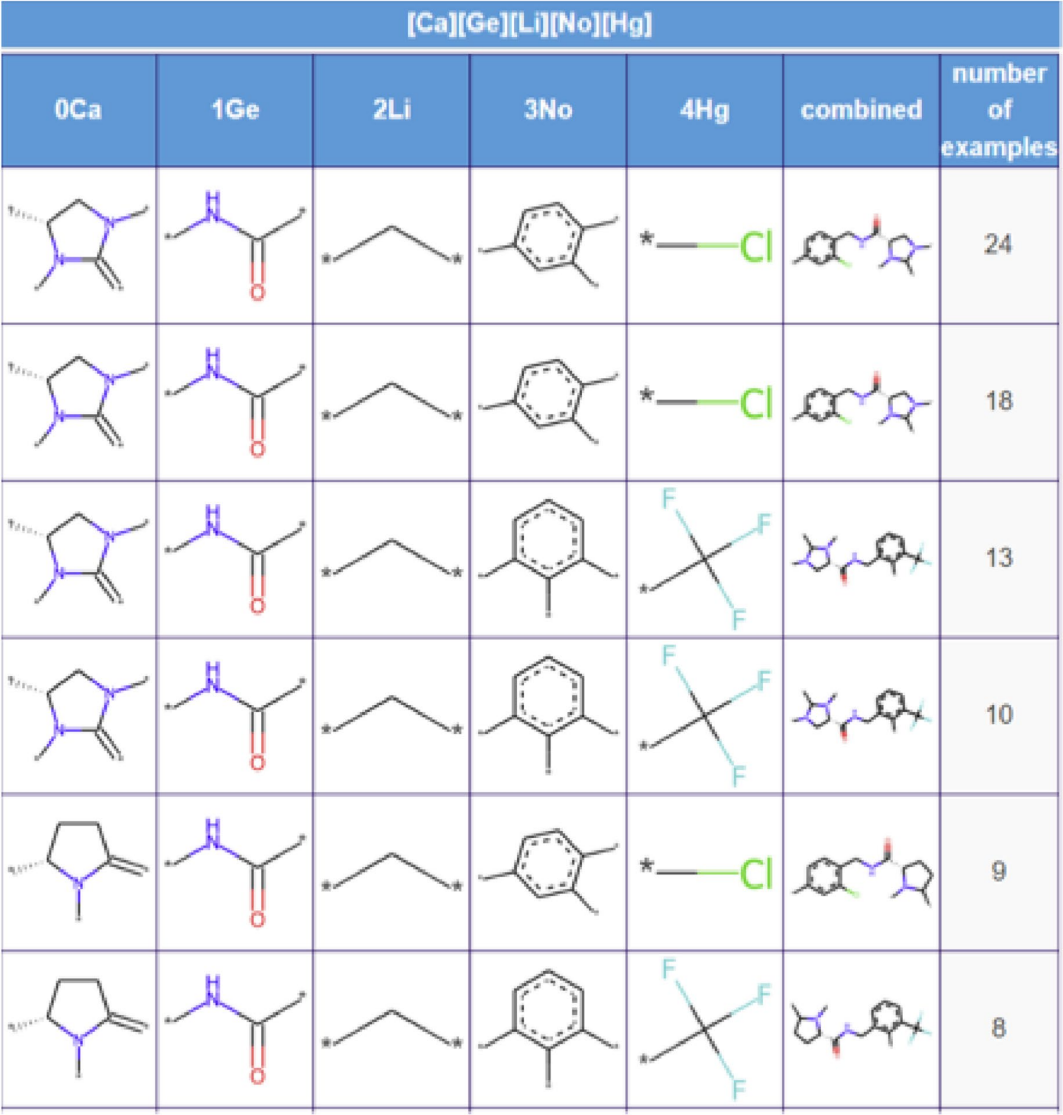
A snippet of the table representation of the RG core shown in Fig. 4

An RG core can also be represented as a table. An extract of the table for the RG core shown in Fig. 4 is displayed in Fig. 6 where the RG core is shown as a SMARTS expression at the top of the table. Each column represents a different node in the RG core and each row represents a unique set of substructures as they exist in one or more molecules in the series. The sixth column shows the substructures combined into a connected substructure with further substitution positions highlighted which show where those molecules are extended beyond the RG core. The final column indicates the number of molecules that contains that combined substructure. There can be multiple molecules represented by each row since the visualisation is focused on the core only and there can be variation beyond the core. In the table, the columns for each of nodes 1Ge and 2Li represent a single substructure which is common to all molecules, respectively, however, the entries in columns representing nodes 0Ca, 3No and 4Hg vary, either by substitution pattern or by substructure.

### Implementation

**Fig. 7:**
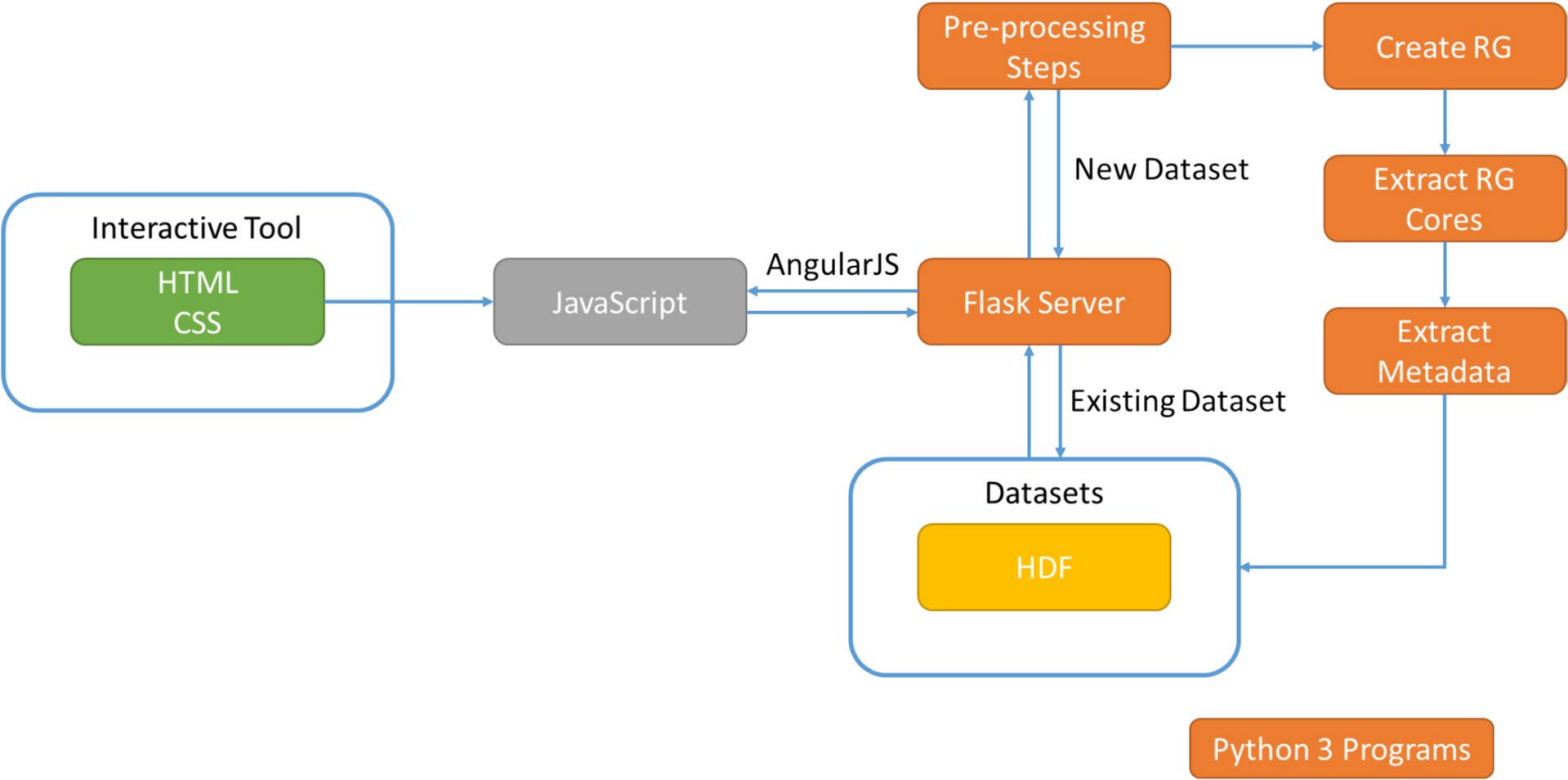
Engineering of the visualisation tool

The visualisation is an interactive tool implemented via a combination of Python 3, Flask, D3, JavaScript, HTML and CSS and is presented as a webpage. The engineering of the tool is illustrated in Fig. 7. The user can view a pre-processed dataset in which case the data is extracted from the corresponding hierarchical data format (HDF) file. The user can also import a new dataset as a set of SMILES strings with a corresponding IDs and pIC50 values. In this case, some pre-processing steps are executed to remove duplicates and to check for valid SMILES, these are then followed by creating the RGs, extracting the RG cores and then extracting the metadata to display within the RG visualisation.

## Methods

### Generating RGs

**Fig. 8:**
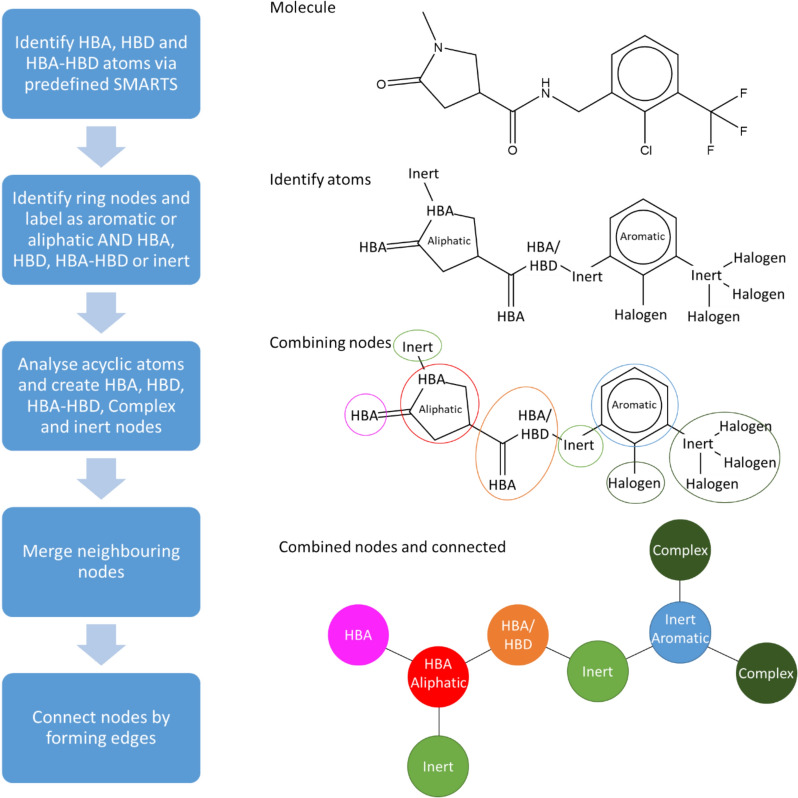
The workflow used to generate RG representations is shown on the left with an example on the right

Figure 8 shows the workflow to generate a RG from a molecule. First, functional groups are identified as hydrogen bond donor (HBD); hydrogen bond acceptor (HBA); or both hydrogen bond donor and acceptor (HBA-HBD), based on user-defined SMARTS (SMILES Arbitrary Target Specification) [19] which are provided via an input file. The SMARTS definitions used for the examples shown here are as follows (HBA: [$([!#6; + 0]);!$([F,Cl,Br,I]);!$([o,s,nX3]);!$([Nv5,Pv5,Sv4,Sv6])]; HBD: [!#6;!H0]). A functional group is identified as HBA-HBD if both the HBA and the HBD SMARTS patterns are matches. Rings are identified using the smallest set of smallest rings and individual rings are labelled as Aromatic or Aliphatic along with their hydrogen bonding characteristics: inert (i.e. no hydrogen bonding character); HBA; HBD; or HBA-HBD.

The acyclic components of the chemical graph are then analysed. Acyclic functional groups that have been identified as HBD, HBA or HBA-HBD are represented by Acyclic nodes with the appropriate label. Any remaining heteroatoms, including halogen atoms, and any branched carbon atoms form nodes which are labelled as “Complex”. Finally, any acyclic atoms that have not previously been included in nodes are defined as Acyclic inert nodes. Next, adjoining nodes are combined and merged as follows: neighbouring nodes of the same type are combined together to form a single node; a HBD or HBA node next to a HBA-HBD is subsumed within the latter node; Acyclic inert nodes connected to one or more “Complex” nodes are combined into a single node labelled “Complex”.

The final step is to connect the nodes via edges according to the bonds in the original structure. A pair of nodes is connected by a single edge unless the two nodes represent fused rings when they are connected by two edges.

**Fig. 9:**
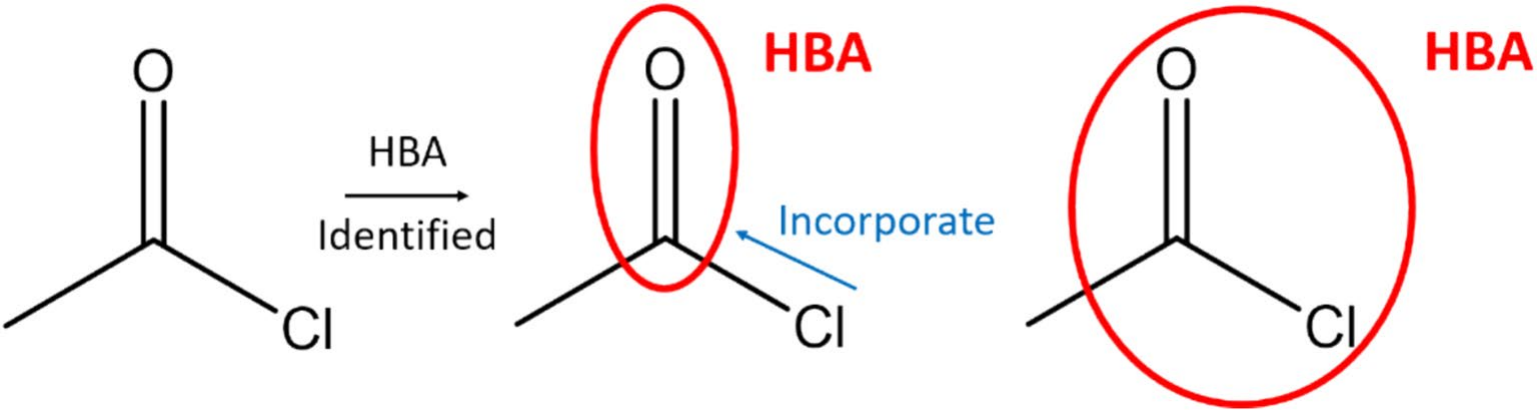
Merging of halogens into HBA nodes

Two additional rules have been implemented. The first is the handling of carbonyl groups; both the carbon and oxygen are considered as a single HBA node unless the carbon atom is within a ring, in which case the oxygen is considered on its own and forms an Acyclic HBA node distinct from the ring node. The second is that a halogen next to a HBA group is combined into the HBA node so that, for example, acyl chlorides form a single node, as shown in Fig. 9. The latter rule was implemented for completeness, since although acyl halides are not expected to occur in LO series due to their reactivity, some were encountered during testing of the methods on ChEMBL.

The RG nodes are labelled using elements not typically found in organic molecules as shown in Table 1. This is in keeping with previous publications relating to reduced graphs and allows the RGs to be represented using standard SMILES grammar so that they can be processed as molecule-types using the RDKit chemoinformatics toolkit.

**Table 1:** The mapping of reduced graph nodes to heavy atoms

Node definition	SMILES code
Acyclic inert	Li
Acyclic HBA	Ga
Acyclic HBD	Gd
Acyclic HBD-HBA	Ge
Aromatic inert	No
Aromatic HBA	Na
Aromatic HBD	Nd
Aromatic HBA-HBD	Ne
Aliphatic inert	Co
Aliphatic HBA	Ca
Aliphatic HBD	Cd
Aliphatic HBD-HBA	Ce
Complex	Hg

As indicated above, each node is also annotated with a SMARTS string that represents the corresponding substructure, with the attachment points being labelled as wild atoms. For example, a phenyl ring with a single connection point is represented by a node labelled as No and annotated by *c1ccccc1.

### Generating RG cores

**Fig. 10:**
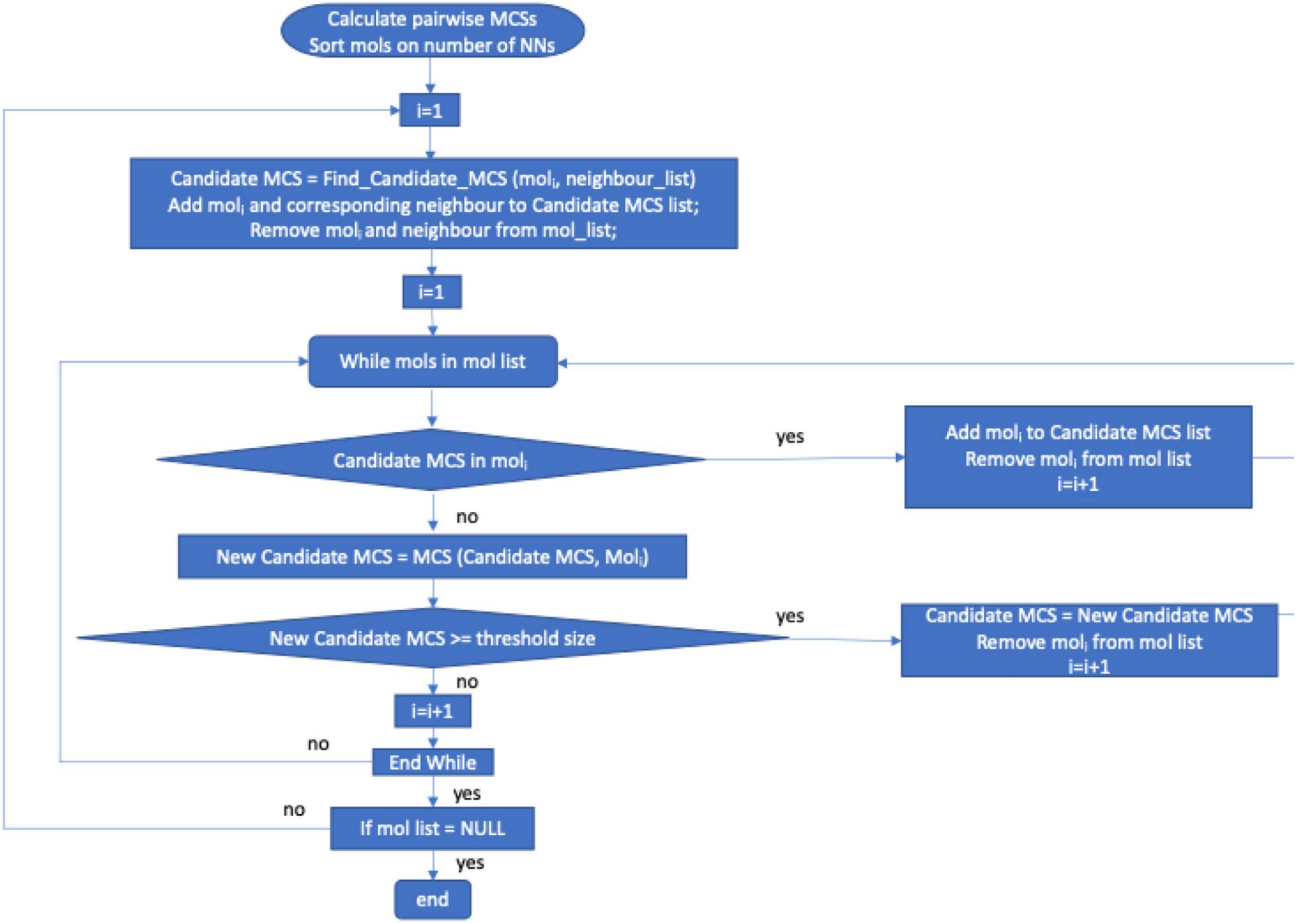
Flowchart to generate RG cores. The Find_Candidate_MCS function starts with the most distant neighbour of the given mol and determines the MCS. If the MCS is greater than or equal to the threshold size then the algorithm proceeds with this. If not, the other neighbours are tried in turn, until an MCS that is larger than the threshold size is found. If none is found then the neighbours are considered in turn and the largest MCS is retained as the Candidiate_MCS

The method for generating the RG cores for a dataset is similar to that described by Gardiner et al. and is based on iteratively calculating MCSs between pairs of RGs [20]. Figure 10 shows a flowchart of the algorithm. There are two user defined parameters: a similarity threshold which is used to determine the near neighbours of each RG; and the minimum RG core size which is the minimum number of nodes that an RG core should have. The similarity between two RGs, A and B, is calculated using the graph variant of the Tanimoto coefficient [21]:

$$T = \frac{MCS}{{A + B - MCS}}$$

where *A* is the number of nodes in RG A; *B* is the number of nodes in RG B; and *MCS* is the number of nodes in the MCS.

The steps to generate the RG cores are as follows:

1. Generate the RG for each of the molecules (which are in arbitrary order). Calculate the pairwise similarity between all RGs in the dataset and, for each RG, store its near neighbours based on the user-defined similarity threshold. Put all molecules into a list.
2. The molecule in the list with the most neighbours is identified as the centroid.
3. Starting with the most distant neighbour of the centroid, find the MCS between the centroid and each neighbour in turn until an MCS is found where the number of nodes is equal to or exceeds the user-defined minimum size. This MCS is then set as the candidate MCS for an RG core, both molecules are removed from the list of molecules, and the algorithm continues to Step 4. If an MCS of the required size is not found, then the largest MCS amongst the neighours is taken as the candidate MCS and that neighbour is removed from the list of molecules.
4. For all other RGs in the molecule list,
  - Compare the RG with the candidate MCS.
  - If the RG contains the candidate MCS, then the RG is associated with the candidate MCS and is removed from the molecule list, and the search moves to the next RG in the list;
    Else if the RG does not contain the candidate MCS then a new MCS is found between the RG and the candidate MCS. The new MCS must be a subgraph of the candidate MCS and the number of nodes in the MCS must be equal to or exceed the user-defined minimum.
    i. If the new MCS passes these requirements, it becomes the new candidate MCS, the molecule (and all previously identified molecules) are associated with the new candidate MCS, the molecule is removed from the molecule list and the search moves to the next RG in the list.
    ii. If the MCS does not pass these requirements, then the candidate MCS is unchanged, the RG is passed over and the search continues.
  - Once all the RGs within the dataset have been searched, the candidate MCS becomes an RG core and is added to a list of RG cores.
5. A check is then made to determine whether all the RGs within the dataset are associated with an RG core, that is, if the molecule list is empty
  - If so, the process stops.
  - If not, then the process is repeated from Step 2.

**Fig. 11:**
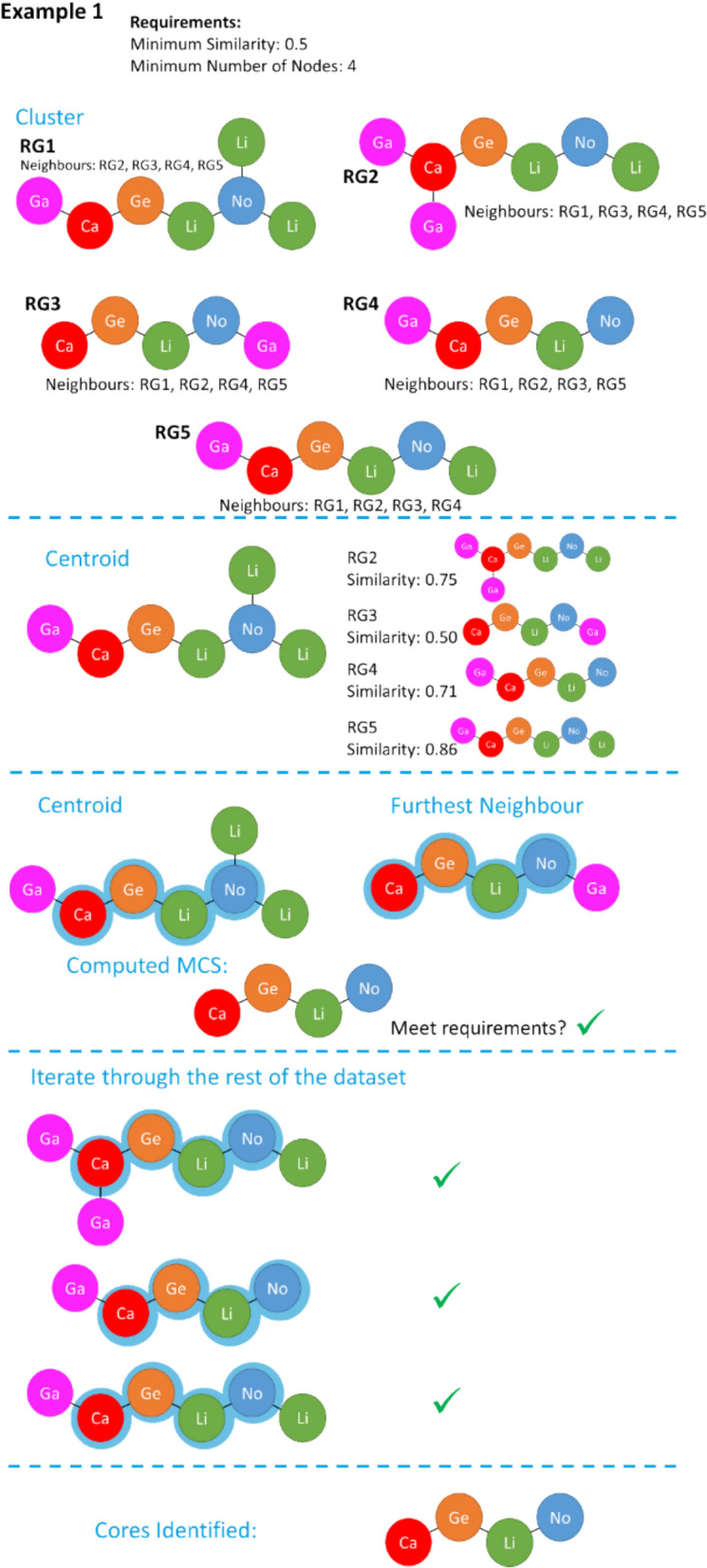
Example of the RG core extraction methodology when the similarity threshold is 0.5 and the minimum number of nodes is 4

**Fig. 12:**
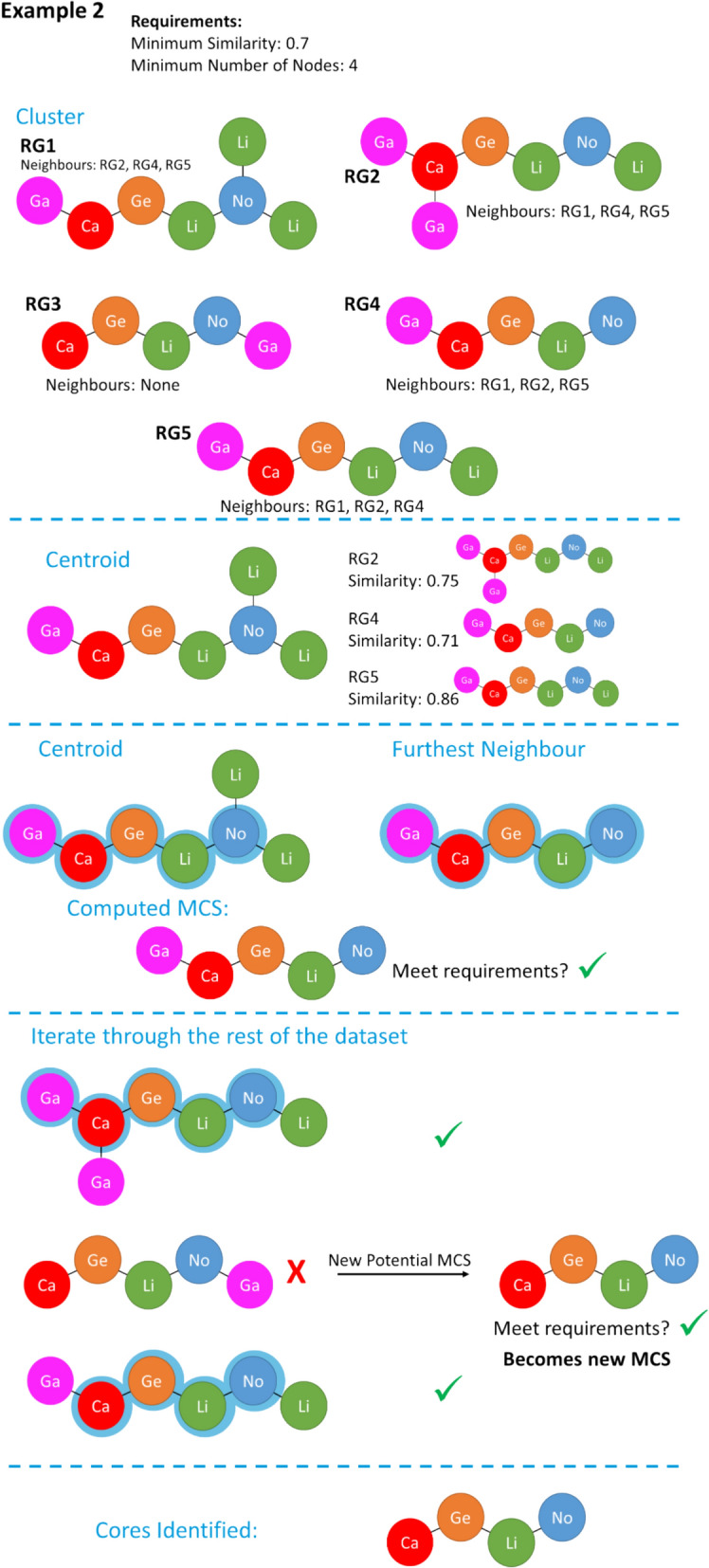
Example of the RG core extraction methodology when the similarity threshold is 0.7 and the minimum number of nodes is 4

Figures 11 and 12 show implementation examples of the workflow. In Fig. 11, all pairs of RGs are within the defined similarity threshold of 0.5 and, therefore, all molecules are in the nearest neighbour (NN) lists of all others. The first molecule is therefore identified as the centroid and its furthest neighbour is determined. The MCS between these two molecules is found and, as it meets the minimum core size requirement of four, the process continues. This MCS is found in the rest of the molecules within the dataset, and the process completes with one RG core identified.

Figure 12 shows the same dataset as in Fig. 11, however, the similarity threshold for determining near neighbours has been increased to 0.7 so that the number of neighbours is no longer the same for all the molecules. Four of the molecules now have three neighbours. The first molecule is chosen as the centroid, as in the previous example. However, a different molecule is now identified as the furthest neighbour and, therefore, a different initial candidate MCS is found. The second molecule contains the MCS and so is added to the list of represented molecules. The third molecule does not and therefore a new MCS is found and becomes the new candidate MCS. The iterations continue using the new candidate MCS, which is found in the remaining molecules. Note that the same RG core is identified in both cases even though the data is processed in different ways, however, this is not always the case when the threshold is varied.

### Assigning molecules to RG cores

Following the identification of RG cores, a second pass is made through the data to ensure that each molecule is associated with all the RG cores that it contains, as described below. This step is needed because a molecule may have been missed due to the order in which they are processed. Following this step, it is possible that a given molecule may be associated with more than one RG core. This step also enables the RG core to be annotated with the underlying substructures and the SAR tables to be constructed by using the mapping procedure described below.

### Mapping molecules to RG cores

**Fig. 13:**
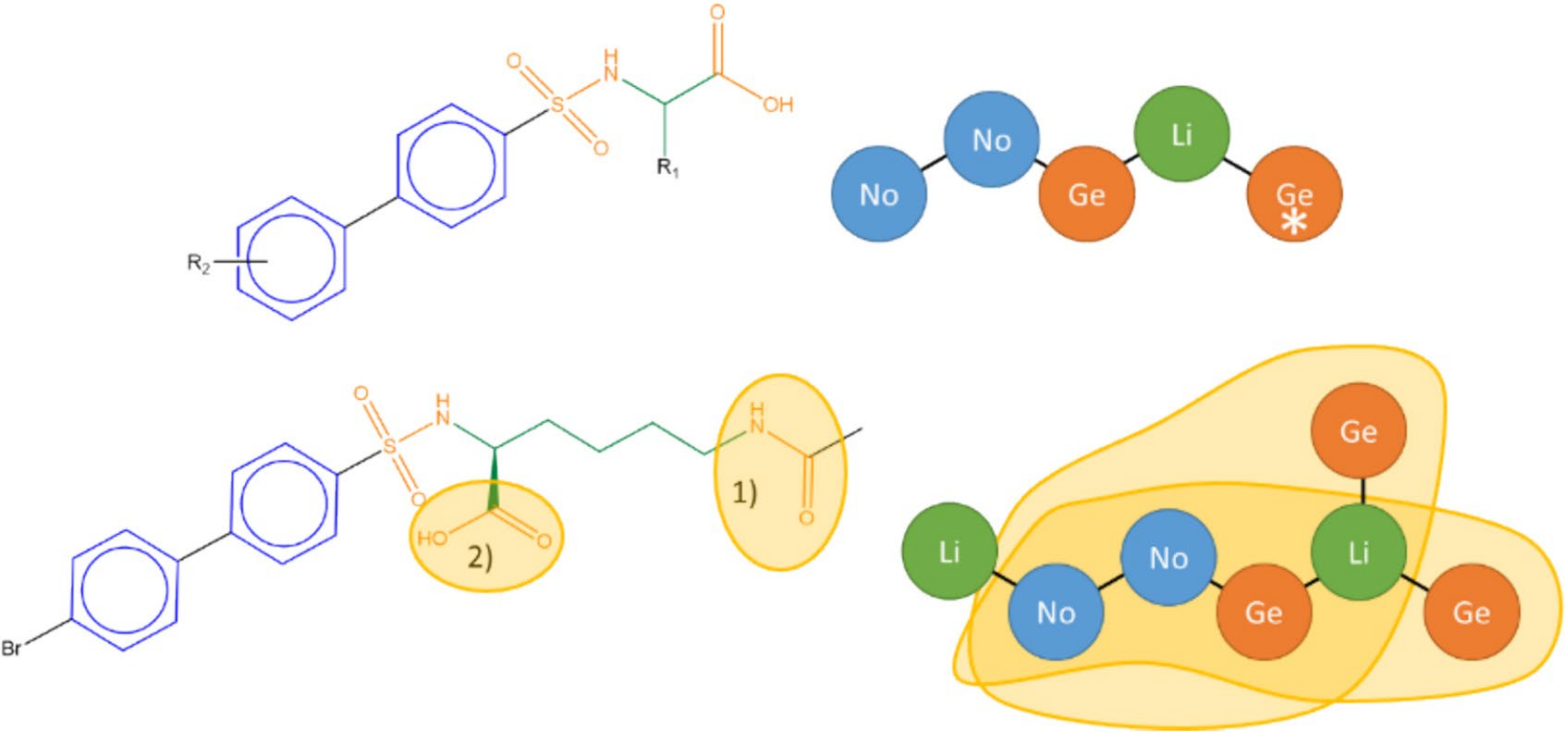
The Markush structure (with R-groups) is shown at the top together with RG core extracted from the dataset. The molecule bottom left has two different mappings to the starred Ge node shown by the highlighted substructures

The mapping of the molecules onto the RG cores is not straightforward since there can be multiple ways to map a molecule. For example, Fig. 13 illustrates a molecule that has two different mappings onto the RG core. The RG core consisting of 5 nodes is shown top right alongside a typical Markush representation of the molecules. A molecule to be mapped is shown below along with its RG representation which has two Ge nodes attached to the right-most Li node. These two Ge nodes represent the substructures labelled as (1) and (2), respectively, on the molecular structure shown bottom left. Two mappings are therefore possible to the RG core, each of which results in a different substructure being associated with the starred Ge node. In the LO dataset from which the example is derived, all the molecules have a terminal COOH group attached to the Li node and hence the most appropriate mapping in this case is the node that represents substructure (2). Thus, in this example it is possible to disambiguate potential mappings by considering all the molecules that map to the RG core. In general, however, the underlying substructures will not be identical and ambiguous mappings are resolved by considering a range of increasingly detailed features of the underlying molecules. The most effective of these is to consider the topological distances between pairs of nodes, as described below where the aim is to identify mappings where the through-bond distances between nodes are similar across the series of molecules represented by the RG core, that is, the substructures represented by the nodes are of a similar size.

For each RG core, a first pass is made through the molecules and all molecules that have only one mapping are mapped to the RG core and the nodes of the RG core are annotated with the corresponding substructures. These unique mappings then form a basis for resolving the mappings of molecules that have multiple ways of being mapped to the RG core. Ambiguous mappings are resolved by calculating *node topological distance maps* and *substituent topological distance maps* for both the RG core and the individual molecules to be mapped, as described below.

**Fig. 14:**
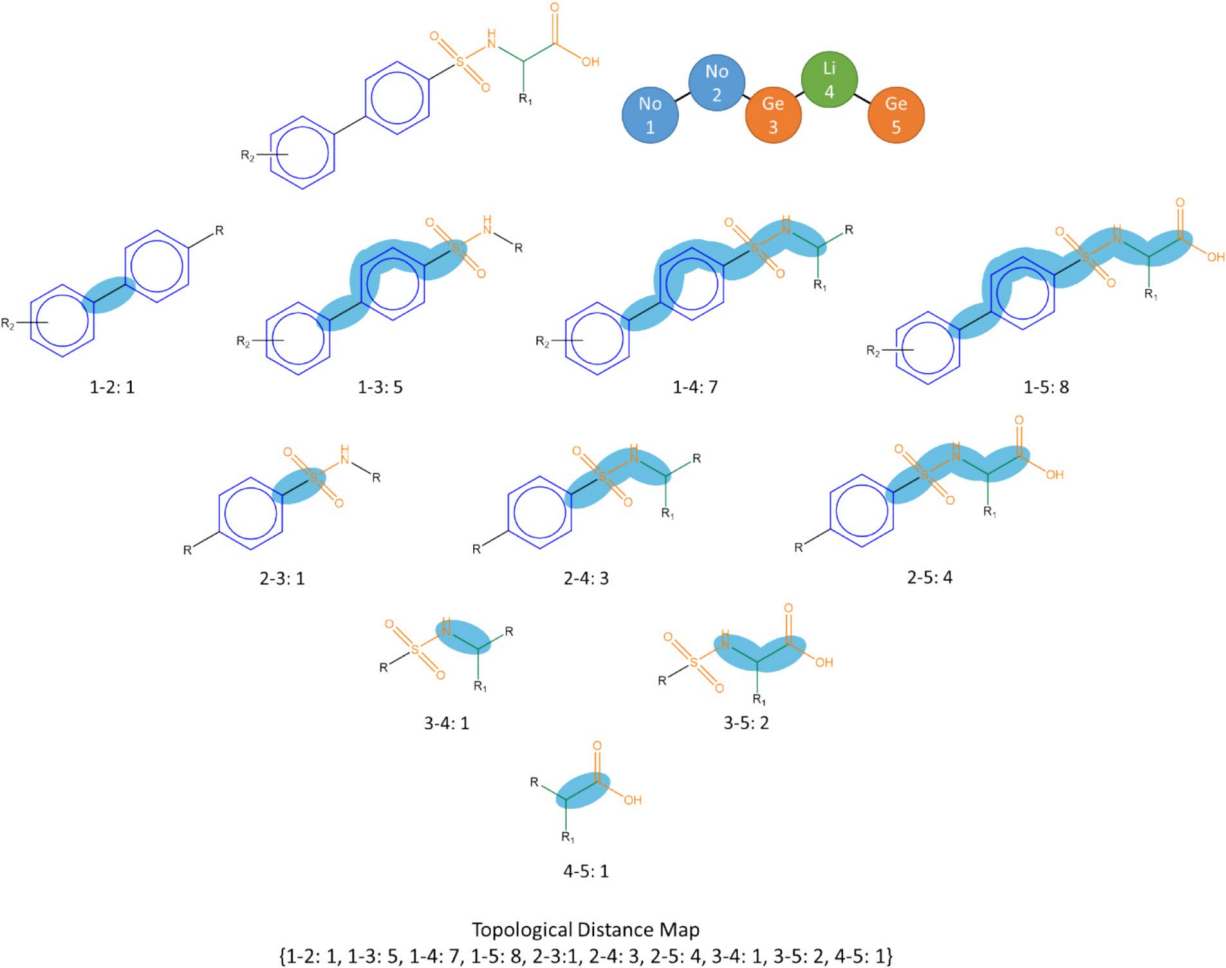
The node topological distance map for an example molecule

First, a node topological distance map is created for an RG core by considering each molecule with a unique mapping, in turn. The node topological distance map consists of all pairs of nodes together with the topological (through-bond) distance between them, which is defined as the shortest bond distance between any pair of atoms in the mapped molecule, where one atom is taken from each node. An example of a node topological distance map is shown in Fig. 14. For example, the shortest bond distance between nodes 1 and 2 is a single bond (and is represented in the topological distance map by 1–2:1), whereas the shortest bond distance between nodes 1 and 4 is seven (and is represented by 1–4:7). If a newly created node topological distance map is the same as an existing one, then it is discarded and the count for the existing map is incremented, otherwise it is appended to form a list of node topological distance maps for the RG core.

**Fig. 15:**
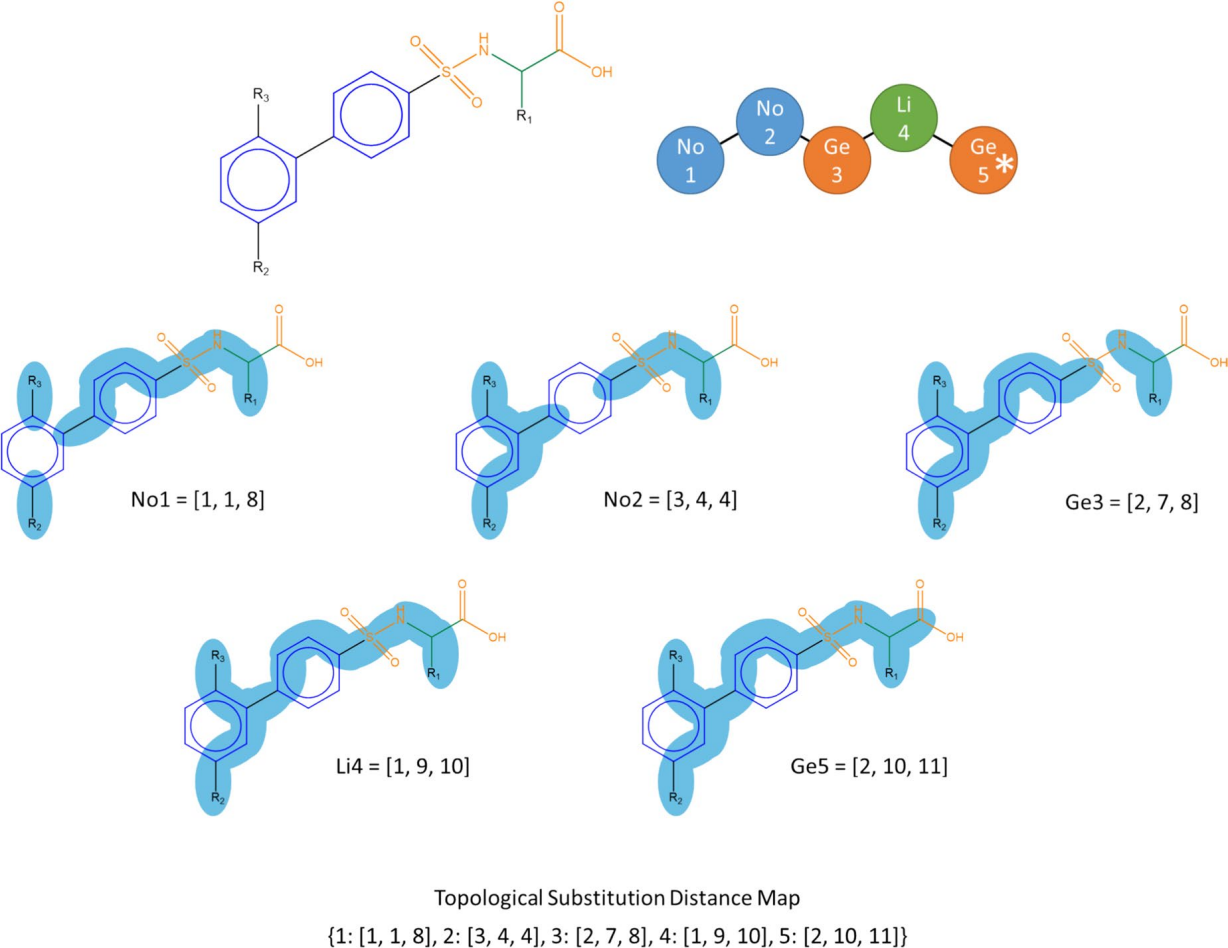
The substituent topological distance map for an example molecule

A substituent topological distance map is also created by finding the shortest topological distance between each node and each substitution site on the RG core. An example of a substituent topological distance map is shown in Fig. 15 where three substituent sites are present indicted by R_1_, R_2_ and R_3_ in the original molecule. These represent parts of the original molecule which are not included within the RG core. For each node, the topological distance to each of the substitution sites is found, for example, for node 2 the through-bond distances to R_1_, R_2_ and R_3_ are 4, 4 and 3, respectively and these are encoded as 2: [3, 4, 4] where the distances are sorted from shortest to longest. As for the topological distance maps, the unique substituent topological distance maps are aggregated (with associated frequency counts) over all molecules with unique mappings to the RG core.

The molecules with ambiguous mappings to the RG core are then considered. For each molecule with multiple mappings, its node topological distance map and substituent topological distance map are calculated and compared with the aggregated maps for the RG core as follows.

If the molecule’s topological distance map is the same as just one of those associated with the RG core then this mapping is used. If there are multiple matches, then the mapping with the largest number of examples in the aggregated map for the RG core is used. If there is more than one such match, then the substituent topological distance maps are compared with the corresponding aggregated substituent topological distance maps for the RG core and similar conditions applied, i.e., if there is a unique match this is chosen, followed by the one with most examples.

The process described thus far is sufficient to resolve the majority of cases, however, additional criteria are applied if required. The complete algorithm for resolving mappings onto the core is shown in the Supporting Information.

## Results and discussion

The methods are demonstrated on a dataset of P2X7 receptor antagonists that represents published lead optimisation studies conducted at GlaxoSmithKline [22]. The full dataset has been deposited in the ChEMBL database [23]. The P2X7 receptor is an ion channel that is activated by ATP and is involved in processes including inflammation and neurological function. The discovery of the P2X7 receptor led to a number of drug discovery programmes and subsequent clinical trials with a resurgence in interest in the target in a diagnostic setting [24].

The dataset consists of 798 compounds obtained from ChEMBL by first downloading the P2X7 human dataset, ChEMBL target ID: CHEMBL4805, and then extracting compounds with the following Document ID’s: CHEMBL1157114, CHEMBL1221272, CHEMBL1268987 and CHEMBL2218064, as these were known to be part of the LO programme carried out at GSK. The compounds were cleaned using the following protocol. Salts were removed using the RDKit function (SaltRemover) which extracts the largest fragment [25]. The molecules were then neutralised using the O’Boyle neutralisation function in RDKit [26]. Molecules without an associated pIC50 value were removed.

The first step was to generate RGs for the compounds. Table 2 shows the average number of atoms in the molecules, the number of unique RGs generated, and the average nodes in the RGs, respectively. The average number of nodes per unique RG is 9.0 and each RG represents, on average, 3.6 molecules.

**Table 2:** The number of molecules, average number of heavy atoms, the number of unique RGs generated, and the average number of nodes per RG

Number of molecules	Average number of heavy atoms per molecule	Number of Unique RG	Average number of nodes in the RGs	Average number of nodes in the unique RGs
798	24.9	245	9.0	9.4

As discussed in the Methods section, the process for generating the RG cores is based on two user-defined parameters: the similarity threshold used to identify near neighbours in the first step of the algorithm; and the minimum number of nodes in an RG core. Table 3 shows the number of RG cores extracted as the similarity threshold is varied between 0.1 and 0.9 and the minimum core size is varied from 2 to 7.

**Table 3:** The number of RG cores extracted as the similarity threshold and the minimum number of nodes are varied

		Minimum RG core size					
		2	3	4	5	6	7
Similarity threshold	0.1	3	5	8	11	17	26
	0.2	3	5	7	9	18	22
	0.3	3	5	8	12	19	30
	0.4	3	6	8	13	20	27
	0.5	3	5	8	13	20	30
	0.6	3	5	8	13	20	33
	0.7	3	5	8	13	20	32
	0.8	3	5	8	12	20	34
	0.9	3	5	8	13	20	42

In general, as the minimum core size increases there is an increase in the number of RG cores found. This is expected because typically the smaller the number of nodes in an MCS the more likely it is to be present in a molecule, for example, an MCS consisting of just two nodes such as an acyclic inert node connected to an aromatic inert node is likely to be present in many molecules. As the minimum core size is increased, the number of molecules that share an MCS is likely to decrease and, therefore, more RG cores are likely to be extracted. Varying the similarity threshold has little effect on the number of RG cores found. A minimum RG core size of five and a similarity threshold of 0.5 were chosen for this analysis which resulted in 13 RG cores.

**Fig. 16:**
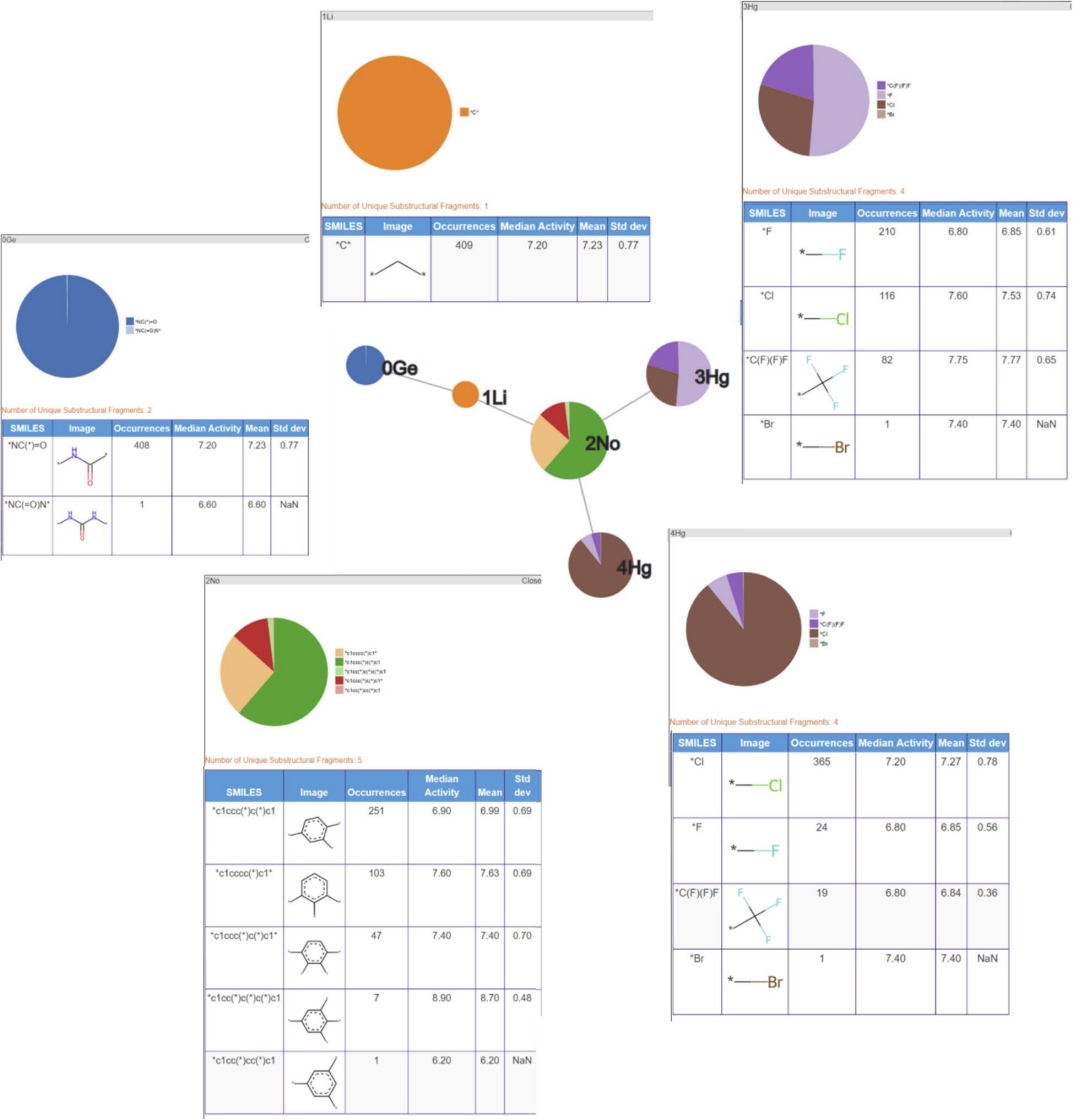
Tabular displays of the substituents represented by each node of RG core 1: [Ge][Li][No]([Hg])[Hg]

The nine most populated RG cores are illustrated in Table 4. The most frequent RG core (RG core 1) represents 409 (> 50%) of the molecules and consists of five nodes which are: an aromatic inert node (labelled No) with two acyclic substituent nodes (Hg) and a further inert acyclic node (Li) which is extended by a HBA-HBD acyclic node (Ge). The underlying substructures represented by each node are shown in Fig. 16. The aromatic node represents a phenyl ring in all the molecules and the five different segments in the node correspond to five different substitution patterns on the ring. The two acyclic nodes labelled Hg each represent halide groups or CF_3_. The third acyclic inert node is a CH_2_ group in all the molecules and is connected to an amide in all but one of the molecules where it represents urea. Further variation in the molecules exists at the substitution positions shown in the substructures, however, the substructures at these positions are not shown as they do not form part of the RG core. Although this RG core represents more than 50% of the molecules in the dataset, it does not include a key part of the molecules which is the heterocycle seen in the example molecule. It is therefore not very informative in terms of understanding the lead optimisation exploration carried out in this dataset. Furthermore, there is a high degree of overlap of the molecules represented by the three most populated RG cores, as show in Table 5.

**Table 4:**
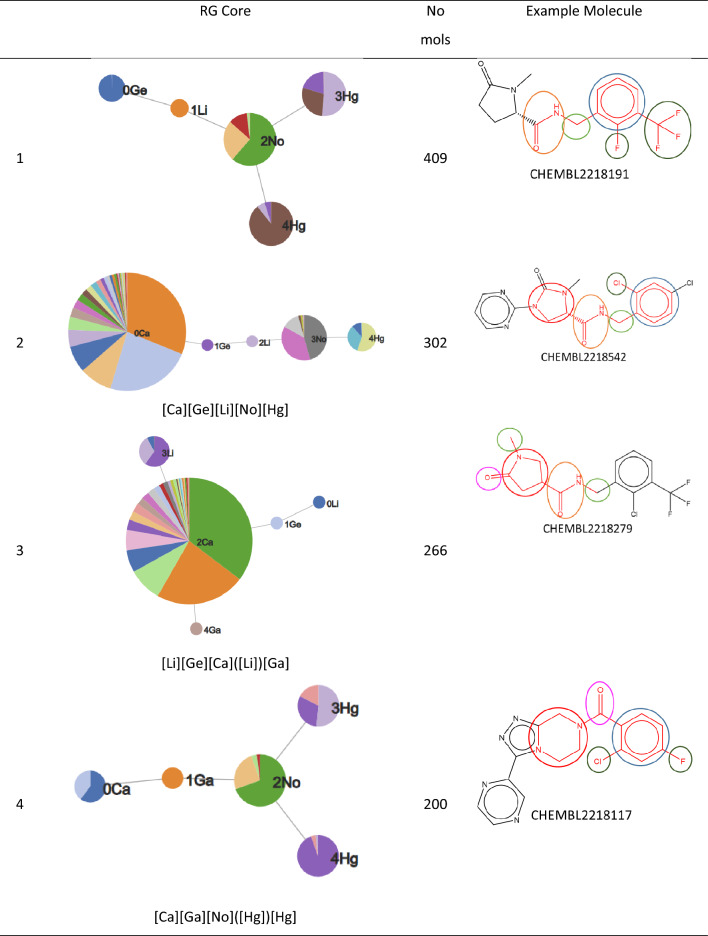

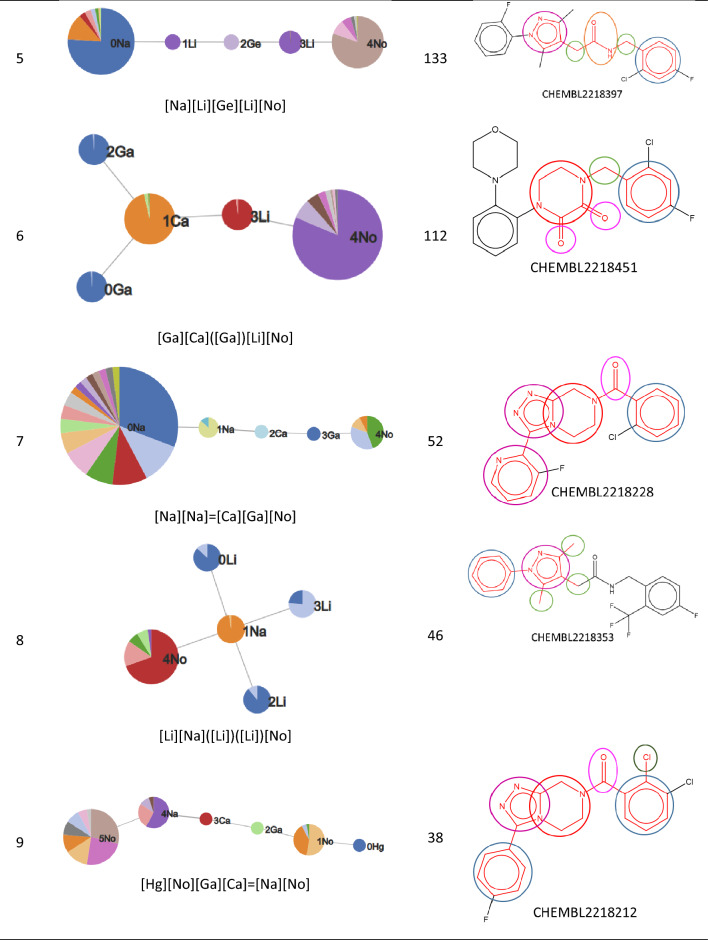
The nine most populated RG cores

The number of molecules represented by each RG core is shown along with an example molecule where the substructures corresponding to nodes in the RG core highlighted

**Table 5:** Overlap of molecules represented by the three most populated RG cores

RG core	1	2	3
1	409	268	228
2	268	302	256
3	228	256	266

RG core 2 is highlighted since this is most representative of the LO series

**Fig. 17:**
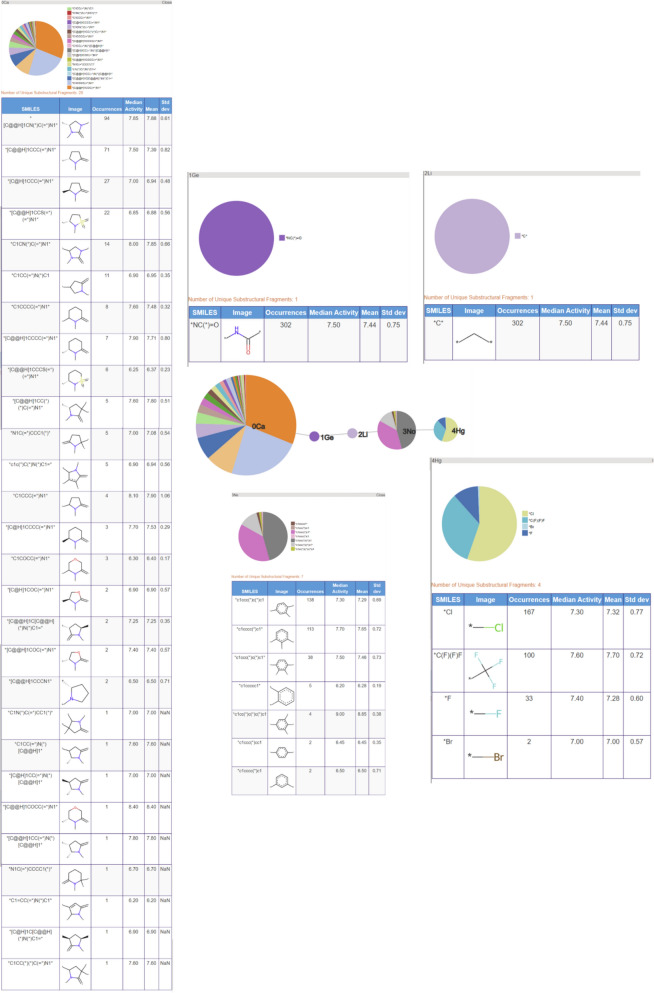
Tabular displays of the substituents represented by each node of RG core 2: [Ca][Ge][Li][No][Hg]

The second most populated RG core also consists of five nodes and represents 302 (38%) of the molecules. It is more informative in terms of capturing the LO series and the motivations behind the data exploration. These molecules are representative of structures derived from pyroglutamic acid amide as explored in Abdi et al. [27]. The underlying substructures explored in the molecules are shown in Fig. 17. Two of the nodes, the central acyclic nodes (Ge and Li), show no substructural variation and represent an acetamide group which is present in all the molecules. The large Ca node (aliphatic HBA ring) indicates that a wide variety of alternative aliphatic ring systems with hydrogen bond acceptor character have been explored. There are 28 distinct rings that include a variety of five and six membered hetero rings which all contain nitrogen with some also containing oxygen or sulphur atoms. The most common ring is imidazole (94 molecules) followed by the pyrrolidine ring (71 molecules). As for the previous RG core, the No node represents a phenyl ring in all the molecules with seven different substitution positions on the ring having been explored. In this case, just one HG node has been identified which represents four different substructures: Cl, Br, F and CF_3_.

**Fig. 18:**
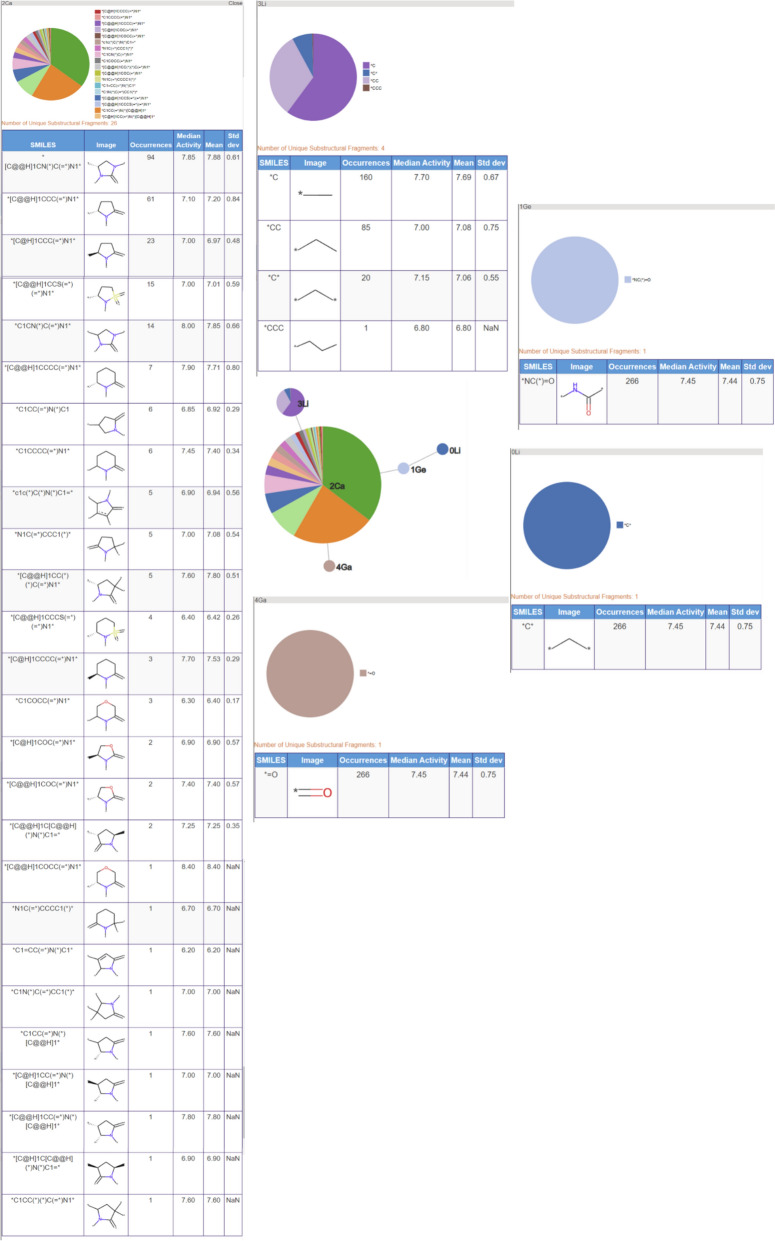
Tabular displays of the substituents represented by each node of RG core 3: [Li][Ge][Ca]([Li])[Ga]

The third most populated RG core (266 molecules) is related to the previous two but is focused on the central acetamide group and the heterocyclic ring; it does not capture the phenyl ring identified in the previous two RG cores. The substructures represented by each of the nodes are shown in Fig. 18. Most variation is seen for the aliphatic HBA ring node and many of the same substructures are present as seen for RG core 2.

The close relationship between the three most populated RG cores is evident when looking at the overlap in the molecules represented by each RG core, Table 5. All three RG cores consist of five nodes all of which contain the central acetamide group, however, RG core 2 is most representative of the series as it captures both the phenyl ring and the aliphatic HBA ring. The molecules represented by RG Core 1 and RG Core 3 which are not represented by RG Core 2, reveal some common substituent patterns on the phenyl ring and the aliphatic heterocycle, respectively.

**Fig. 19:**
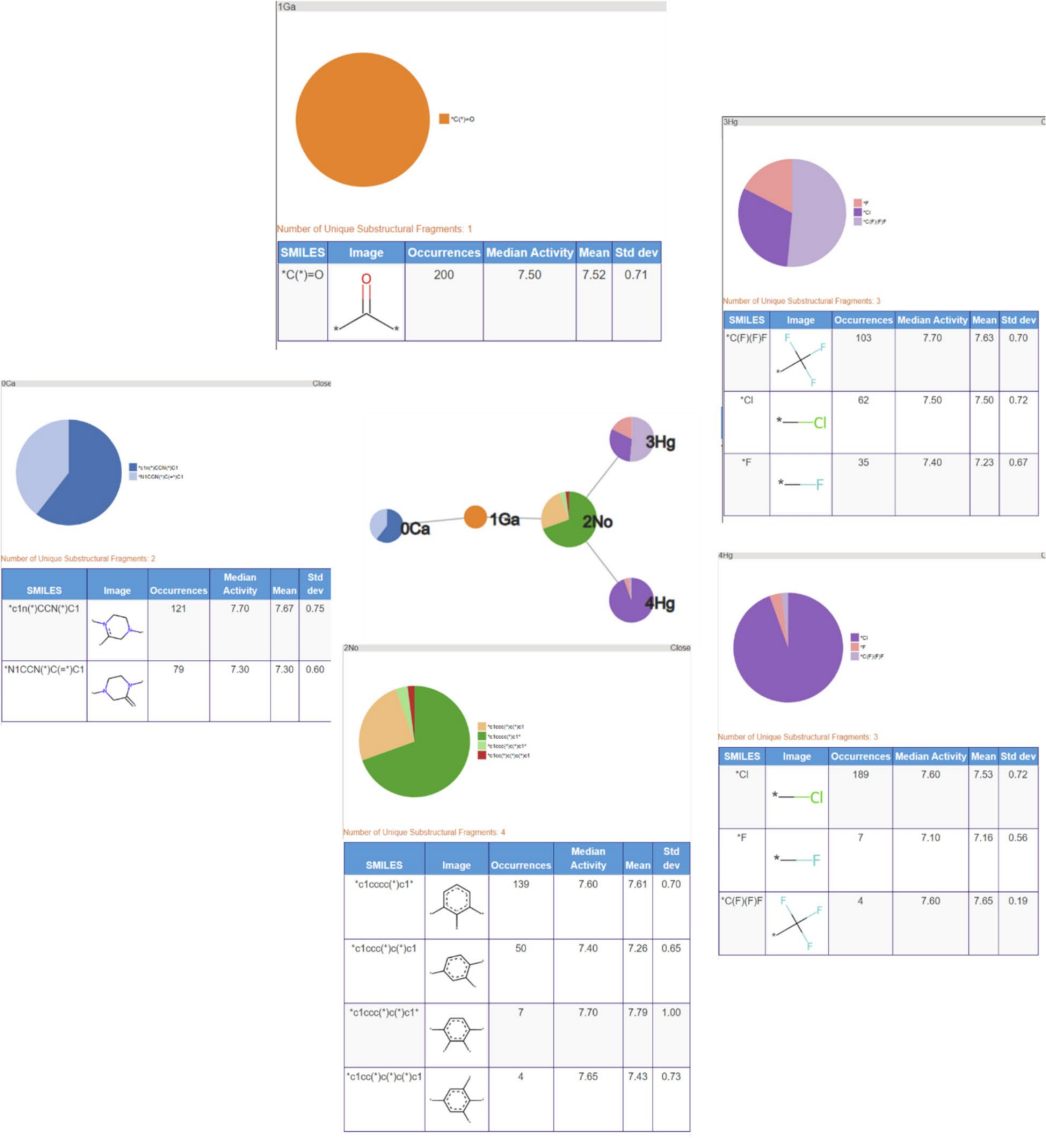
Tabular displays of the substituents represented by each node of RG core 4: [Ca][Ga][No](Hg])[Hg]

**Fig. 20:**
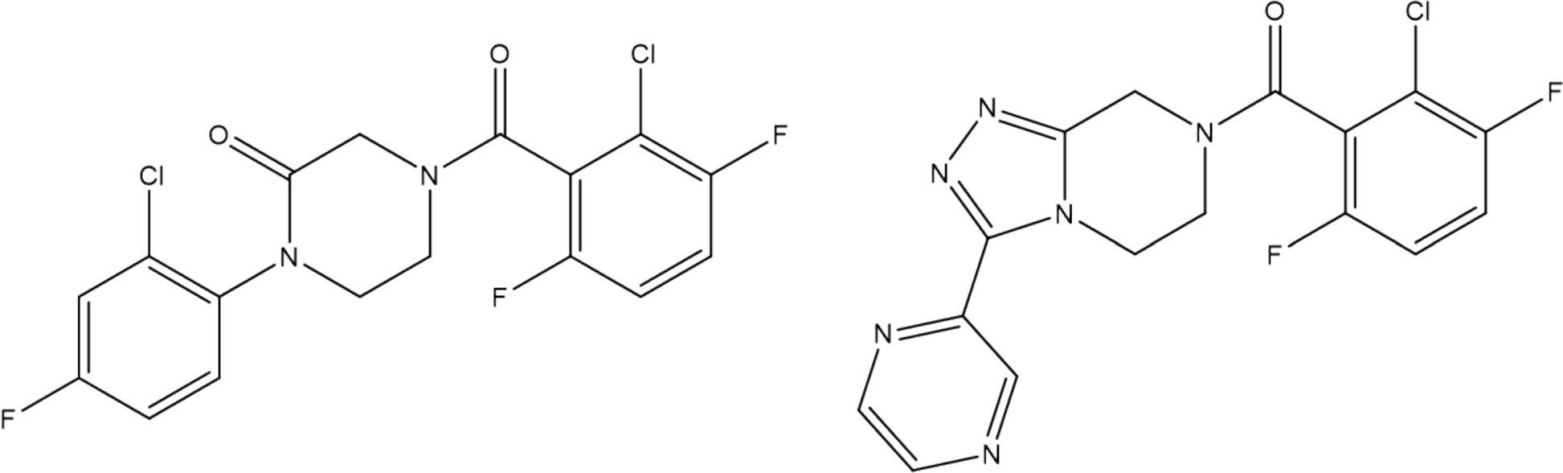
Example molecules which show the two different aliphatic heterocycles represented by the RG core in Fig. 19

The fourth most populated RG core represents compounds where the acetamide central group has been replaced by a carbonyl group, which is represented by a single node (Ge) in the RGs. Although a relatively large number of molecules is represented by this RG core (200), there are just two variants of the aliphatic heterocycle (Fig. 19) with example molecules for each variant shown in Fig. 20. The triazolopiperidines are the larger set (121 of 200) (on the right of Fig. 20) and they include compounds from the series reported by Dean et al. [28]. The piperazinones are the smaller set (79 of 200) (on the left of Fig. 20) and they include compounds from the series reported by Chambers et al. [29]. In this case, the RG core represents two different series of compounds which have been reported in two different patents.

**Fig. 21:**
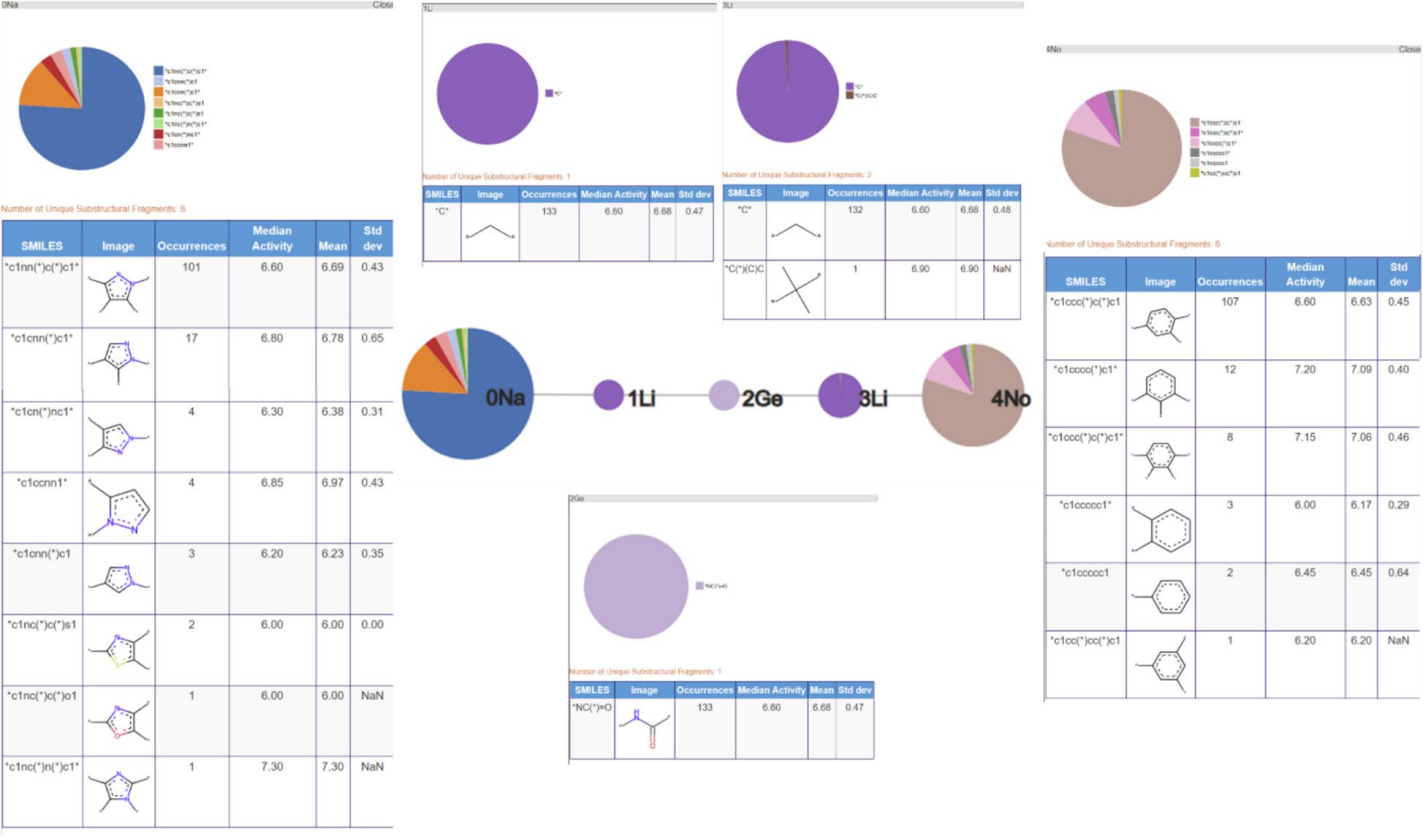
Tabular displays of the substituents represented by each node of RG core 5: [Na][Li][Ge][Li][No]]

The fifth RG core represents 133 compounds, as shown in Fig. 21. For this series, the central amide group is extended by a carbon on both sides and the heterocycle is now an aromatic HBA ring, rather than an aliphatic heterocycle. These compounds correspond to the 1H-pyrazol-4-yl) acetamides described Chambers et al. [22]. The aromatic heterocycle node represents eight different substructures that are all five membered rings consisting of different nitrogen, oxygen and sulphur derivatives. The aromatic inert ring represents a phenyl ring (as in the previous two series) with six different substitution patterns.

**Fig. 22:**
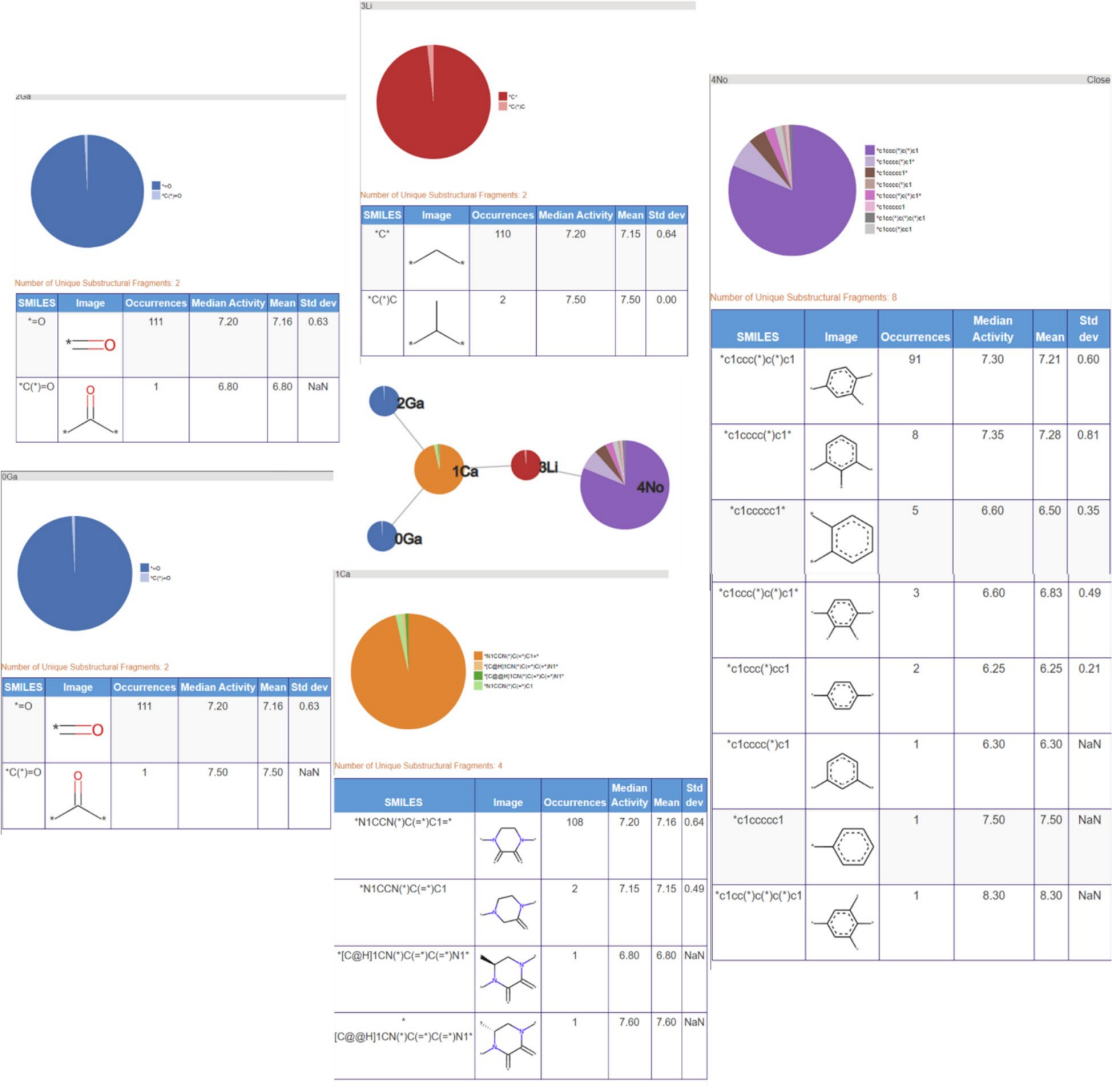
Tabular displays of the substituents represented by each node of RG core 6: [Ga][Ca]([Ga])[Li][No]

The sixth RG core, Fig. 22, represents 112 molecules where the group linking the phenyl and heterocycle is now either a methylene (110 molecules) or an ethylene (2 molecules) group. All the molecules associated with this RG core are reported in the Supplementary bioactivity data of Chambers et al. [22].

The seventh RG core represents 52 compounds, however, the majority of these molecules (44 molecules) are also represented by RG core 4. They contain the triazolopiperidines as covered by RG core 4 but the RG core has been extended to include a further heterocyclic node which is attached to the fused system. The extended node represents a variety of aromatic heterocycles.

The eighth RG Core represents 46 molecules of which 42 are also represented by RG Core 5, i.e., they are a subset of the 1H-pyrazol-4-yl) acetamides described Chambers et al. [22]. In this case, the RG Core is focused on just the heterocyclic ring and its substituents and does not include the central amide group or the phenyl ring.

The final RG core shown above represents 38 molecules, of which 36 are already represented by the fourth most populated RG core, i.e. they are part of the carbonyl series.

In summary, the thirteen RG cores that were identified can be grouped according to the overlap of the molecules they represent. For one group (RG cores 1, 2 and 3) the RG cores focus on different aspects of one lead optimisation series. For other groupings, the RG cores represent subsets of compounds identified by other cores, for example RG cores 7 are 9 are largely subsets of the molecules represented by RG core 4; and similarly for RG core 8 which represents a subset of the molecules covered by RG core 5. From this analysis, the most informative RG cores for this dataset are RG Cores 2, 4, 5 and 6 and these four successfully represent the four series of compounds explored by GSK in the extracted dataset.

The tabular displays of the substructures represented by the cores provide further information including the number of molecules that contain each substructure and the mean and median activities of those molecules. These tables allow the user to rapidly view the substructural variation that has been explored at each position in the series and can also give some clues to the structure–activity relationships that exist within a compound series. For example, considering the molecules represented by RG core 2, those containing the R pyrrolidine ring (71 molecules) have median and mean activity of 7.50 and 7.39, respectively, compared to 7.00 and 6.94 for the molecules containing the S pyrrolidine ring (27 molecules). By comparing across RG cores, it is immediately obvious that there has been less exploration of the heterocycle in RG core 5 relative to RG core 2 or RG core 6, so this could be an area of future focus.

The method presented here provides a visual representation that summarises the SAR of compound series. As noted above, the visualisation shows areas of chemistry that have been underexplored or where one substituent has dominated. The fact that the definition of series is automated, allows project teams to review the SAR in an unbiased manner, facilitating a review across all compounds in the project. This is the main intent of the study. A second utility is in helping to frame the output from design workflows, for example, from active learning approaches, where project teams can be provided with a visual representation of the design in the context of the existing data and gain an understanding of why a particular region is being explored over another. A third utility is in the consistent representation of chemical series in an unbiased manner that can be useful in assessment of the predictivity of models on subclasses of compounds.

## Conclusions

A methodology is described for organising compounds in a dataset into compound series, each of which is represented by what is referred to as an RG core. An RG core captures the common elements of the series as connected RG nodes in a similar way to a conventional Markush structure and associated SAR table, however, the use of RG representations allows molecules with different substructures to be brought together, provided that those substructures have the same ring/acyclic structural forms and compatible hydrogen bonding characteristics. We have shown how the approach is able to group together molecules from the same series while taking account of small substructural differences in the compounds. We have also demonstrated the ability to extract different series from a dataset that represents different strands followed in a LO programme. The RG cores can be visualised using graphical elements that help the user to easily see the major points of variability within the compound series. The nodes are represented as pie charts which are sized according to the number of different substructures the node represents. The number of segments also reflects the number of underlying substructures and the size of a segment reflects the number of molecules that contain a particular substructure. The use of pie charts gives the user an immediate impression of the extent to which each region of the molecules has been under- or over-explored. The visualisation tool also allows the user to drill down to view the individual substructures in tabular form along with mean and median activity values of the molecules in the dataset that are represented by a particular substructure.

## Supplementary Information

Supplementary material 1.

## Data Availability

Data and software are available at https://github.com/SheffieldChemoinformatics/reduced_graph_visualisation.
