# Supplementary material for: Visualising lead optimisation series using reduced graphs
Source: J Cheminform. 2025 Apr 24;17:60. doi: 10.1186/s13321-025-01002-7 (PMC12023594; doi:10.1186/s13321-025-01002-7)

**Visualising Lead Optimisation Series Using Reduced Graphs**

Jessica Stacey^1^, Baptiste Canault^2^, Stephen Pickett^2^, Valerie J Gillet^1*^

^1^ Information School, University of Sheffield, The Wave, 2 Whitham Road, Sheffield S10 2AH, UK

^2^ GlaxoSmithKline, Gunnels Wood Road, Stevenage, Herts SG1 2NY, UK

Supplementary Information

The full algorithm for mapping the reduced graph representations of molecules onto an RG core.


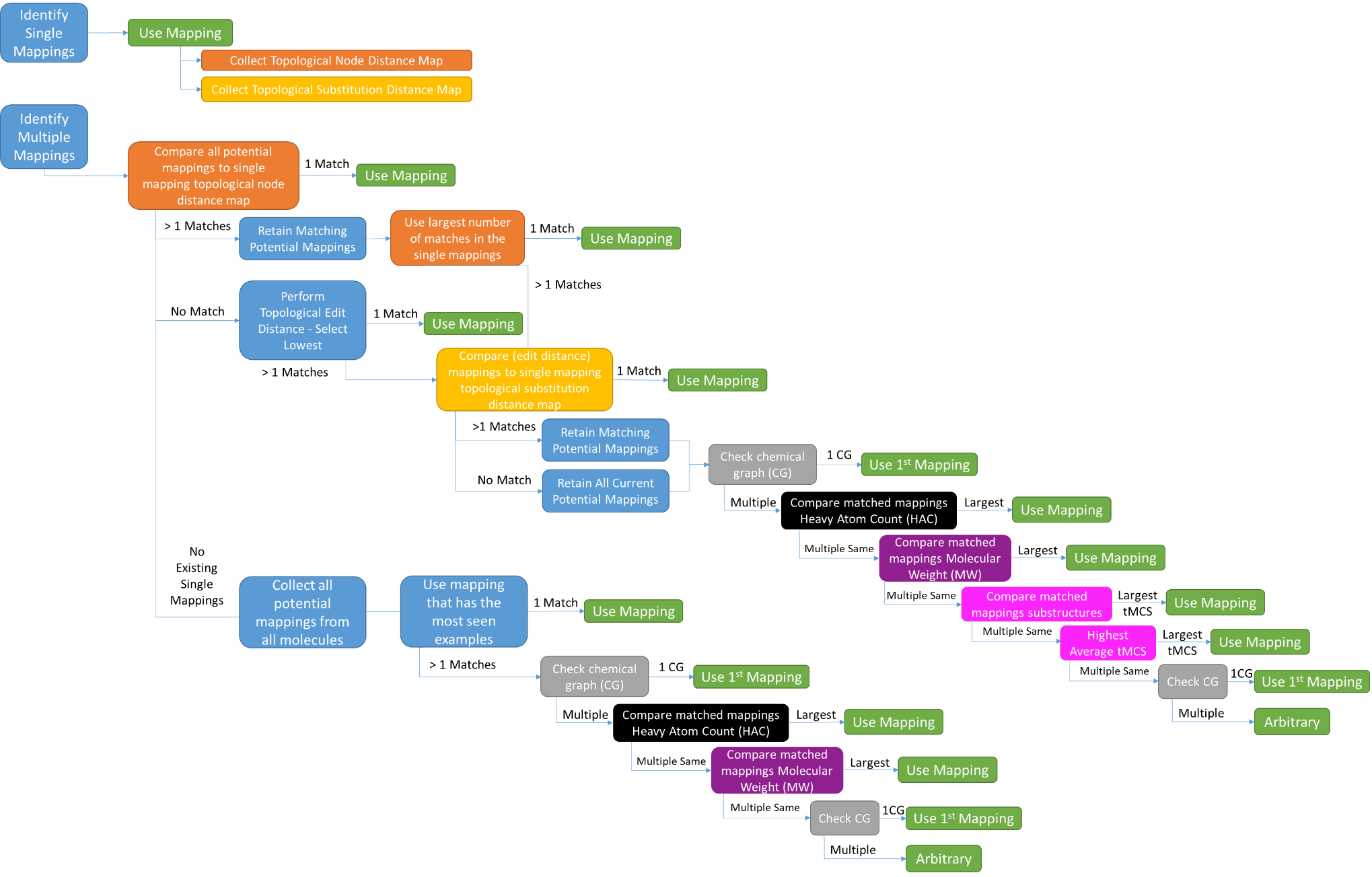

Supplement: Supplementary file 1 — Supplementary material 1. [file 13321_2025_1002_MOESM1_ESM.docx]
